# O‐GlcNAcylation promotes malignancy and cisplatin resistance of lung cancer by stabilising NRF2

**DOI:** 10.1002/ctm2.70037

**Published:** 2024-10-02

**Authors:** Yihan Zhang, Changning Sun, Leina Ma, Guokai Xiao, Yuchao Gu, Wengong Yu

**Affiliations:** ^1^ Key Laboratory of Marine Drugs School of Medicine and Pharmacy Ocean University of China Qingdao China; ^2^ Laboratory for Marine Drugs and Bioproducts Qingdao Marine Science and Technology Center Qingdao China; ^3^ Key Laboratory of Glycoscience & Glycotechnology of Shandong Province Qingdao China

**Keywords:** drug resistance, lung cancer, NRF2, O‐GlcNAc, ROS

## Abstract

**Background:**

The transcription factor NRF2 plays a significant role in regulating genes that protect cells from oxidative damage. O‐GlcNAc modification, a type of posttranslational modification, is crucial for cellular response to stress. Although the involvement of both NRF2 and O‐GlcNAc in maintaining cellular redox balance and promoting cancer malignancy has been demonstrated, the potential mechanisms remain elusive.

**Methods:**

The immunoblotting, luciferase reporter, ROS assay, co‐immunoprecipitation, and immunofluorescence was used to detect the effects of global cellular O‐GlcNAcylation on NRF2. Mass spectrometry was utilised to map the O‐GlcNAcylation sites on NRF2, which was validated by site‐specific mutagenesis and O‐GlcNAc enzymatic labelling. Human lung cancer samples were employed to verify the association between O‐GlcNAc and NRF2. Subsequently, the impact of NRF2 O‐GlcNAcylation in lung cancer malignancy and cisplatin resistance were evaluated in vitro and in vivo.

**Results:**

NRF2 is O‐GlcNAcylated at Ser103 residue, which hinders its binding to KEAP1 and thus enhances its stability, nuclear localisation, and transcription activity. Oxidative stress and cisplatin can elevate the phosphorylation of OGT at Thr444 through the activation of AMPK kinase, leading to enhanced binding of OGT to NRF2 and subsequent elevation of NRF2 O‐GlcNAcylation. Both in cellular and xenograft mouse models, O‐GlcNAcylation of NRF2 at Ser103 promotes the malignancy of lung cancer. In human lung cancer tissue samples, there was a significant increase in global O‐GlcNAcylation, and elevated levels of NRF2 and its O‐GlcNAcylation compared to paired adjacent normal tissues. Chemotherapy promotes NRF2 O‐GlcNAcylation, which in turn decreases cellular ROS levels and drives lung cancer cell survival.

**Conclusion:**

Our findings indicate that OGT O‐GlcNAcylates NRF2 at Ser103, and this modification plays a role in cellular antioxidant, lung cancer malignancy, and cisplatin resistance.

## Graphical abstract

• Oxidative stress activates AMPK to phosphorylate OGT, which enhances the binding of OGT with NRF2 and increases the O‐GlcNAcylation of NRF2.

• O‐GlcNAcylation of NRF2 at Ser103 inhibits the binding of KEAP1 to NRF2 and then promotes its stability, nuclear localisation and transactivation.

• O‐GlcNAcylation of NRF2 promotes malignancy and confers cisplatin resistance in lung cancer.

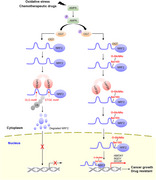

## 1 INTRODUCTION

Among all types of cancer, lung cancer is the primary reason for global mortality. The main objective of lung cancer research is to comprehend and recognise new pathways that can be targeted for therapy. The recurrence caused by inherent and/or acquired treatment resistance is the main obstacle to curing lung cancer.^1^

Reactive oxygen species (ROS) imbalance is closely linked to certain physiological as well as pathological conditions, such as aging, hypertension, ischemic heart disease, insulin resistance, inflammation and cancer.^2^, ^3^, ^4^, ^5^, ^6^ ROS can originate from sources inside or outside the cell,^7^ and maintaining its balance is crucial for the cell viability and proper cell communication.^8^ For cancer, studies have found that increased ROS activates pro‐tumour signals, drives DNA damage as well as genetic instability, enhances cell survival and proliferation, and induces immunosuppressive microenvironment.^9^ Nevertheless, an overabundance of ROS, triggered by oxidative stress in traditional treatments like chemotherapy and radiotherapy, leads to the demise of cancer cells.^10^ Therefore, cytoprotective antioxidation‐induced drug resistance poses a significant challenge to cancer treatment.

Nuclear transcription factor erythroid 2p45 (NF‐E2)‐related factor 2 (NRF2) is the transcription factor responsible for managing oxidative stress, overseeing inflammation, preserving mitochondrial function, and maintaining protein balance within cells.^11^, ^12^, ^13^ It plays a role in controlling various cytoprotective genes associated with antioxidant response mechanisms, including glutathione (GSH) production, removal of ROS, drug transport, and detoxification of xenobiotics.^14^, ^15^, ^16^ Given the pivotal role of ROS equilibrium in oncogenesis, it is not unexpected that persistent activation of NRF2 is frequently observed in human malignancies as well as correlated with resistance to chemotherapy in well‐established neoplasms.^17^ Therefore, revealing the regulatory mechanism of NRF2 holds promise in elucidating the mechanisms underlying cancer development and developing alternative treatment strategies.

NRF2's expression and function are carefully controlled through various mechanisms, particularly through post‐translational modifications. Typically, NRF2 function is usually controlled by interacting with its inhibitor Kelch‐like ECH‐associated protein 1 (KEAP1), a molecule sensitive to redox and acts as a substrate adaptor for the E3 ubiquitin ligase, controlling the ubiquitination and degradation of NRF2.^18^ Upon oxidative stress, the conformation of KEAP1 is changed, losing the binding ability to NRF2, releasing NRF2 into the nucleus to exert its antioxidant effect.^19^, ^20^, ^21^ Subsequent studies found that the key autophagy factor p62/sequestosome 1 (SQSTM1) can sequester KEAP1 into autophagosomes and promote NRF2 activation.^22^, ^23^ In addition to ubiquitination, NRF2 can also undergo various post‐translational modifications, including phosphorylation,^24^, ^25^, ^26^ acetylation^27^, ^28^ and methylation,^29^ which play a crucial role in NRF2's regulation. Although NRF2 regulation has been widely reported, there remains a need to further investigate other post‐translational modifications (PTMs), which could further clarify the NRF2's role in cancer occurrence and treatment response.

O‐linked β‐N‐acetylglucosamine (O‐GlcNAc) modification is a dynamic and reversible post‐translational modification on protein serine or threonine residues.^30^ N‐acetylglucosamine transferase (OGT) and N‐acetylglucosidase (OGA) are involved in the addition and removal of N‐acetylglucosamine, respectively.^31^, ^32^ O‐GlcNAcylation is involved in regulating protein function through different mechanisms, including protein‐protein interaction, protein expression or degradation and protein localisation.^33^, ^34^ Numerous proteins has been detected with O‐GlcNAcylation, which play an important role in cell signaling, gene expression, cellular energy utilisation, and various biological activities.^35^, ^36^ Consequently, the dysregulated OGT/O‐GlcNAcylation cascade is involved in the pathogenesis of numerous disorders, encompassing cardiovascular diseases,^37^ diabetes mellitus,^38^ neurological ailments,^39^ and even malignancies.^40^, ^41^, ^42^, ^43^ Increased O‐GlcNAcylation levels were detected in various types of diverse malignancies, including breast, esophageal, colon and lung cancers.^41^, ^44^, ^45^, ^46^ Studies have found that O‐GlcNAcylation, as an intracellular ‘nutrition sensor’, is closely related to oxidative stress, but the mechanism remains unclear.^47^, ^48^

Here, we studied the O‐GlcNAcylation of NRF2 and its roles and mechanisms in oxidative stress lung cancer malignancy. We investigated the possible function and molecular mechanism of NRF2 O‐GlcNAcylation in tumour cell survival and chemoresistance both in vitro and in vivo. Our research suggests a promising strategy for combating cisplatin resistance in lung cancer patients.

## 2 MATERIALS AND METHODS

The details of Materials and Methods are provided in the Supplementary Information.

### 2.1 Cell culture

All cells were cultured with 10% FBS (PAN Biotech, #ST30‐3302). HEK293T and A549 cells were cultured in DMEM (HyClone, #SH30243.01) medium. NCI‐H1299 cells were cultured in RPMI‐1640 (HyClone, #SH30027.01) medium.

### 2.2 Establishment of cell lines

Lentiviral transduction was performed as described below. HEK293T cells were simultaneously transfected with pMD2.G (Addgene, #12259), psPAX2 (Addgene, #12260), and either pLKO.1 shRNA or pLV cDNA constructs by utilising PEI transfection reagent (Sigma‐Aldrich). Cells were viral infected for 48 h, followed by selection using hygromycin B (200 µg/mL, Shandong Sparkjade Bio‐technology Co., Ltd., #SJ‐MA0066) for pLKO.1 clones or puromycin (2 µg/mL, Shandong Sparkjade Bio‐technology Co., Ltd., #SJ‐MA0040) for pLV clones for 1−2 weeks. OGT shRNA targeting sequence: CTTTAGCACTCTGGCAATTAA.

### 2.3 Immunoprecipitation (IP) and Co‐IP assay

To conduct IP assay, the cells were disrupted in RIPA buffer containing 40 mM GlcNAc, 10 µM PUGNAc (MCE, #HY‐108241), 2 mM STZ (MCE, #HY‐13753), 10 µM Thiamet G (MCE, #HY‐12588), and full protease inhibitors (TargetMol, #C0001). To conduct the Co‐IP test, cells were disrupted in NP40 lysis solution containing 40 mM GlcNAc, 10 µM PUGNAc, 2 mM STZ, 10  µM Thiamet G, and full protease inhibitors for a duration of 20 min while kept on ice. After centrifuging the soluble part of the supernatant, leave 50 µL of supernatant as input. After incubating the lysate mixtures with specified primary antibodies and either protein A/G magnetic beads (Merck Millipore, #LSKMAGA) or protein A/G agarose (Santa Cruz, #sc‐2003) overnight. Subsequently, the beads wash five times with NP40 lysis buffer and were detected by immunoblotting. Additional antibody details are provided in the supplementary materials.

### 2.4 Immunoblotting

Cells were disrupted using RIPA buffer containing 40 mM GlcNAc, 10 µM PUGNAc, 2 mM STZ, 10  µM Thiamet G, and full protease inhibitors. After being separated through SDS‐PAGE, the proteins were then transferred to the PVDF membrane. The designated antibodies were left to incubate at 4 °C overnight, followed by a 1‐h incubation of HRP‐labelled secondary antibodies after being washed three times with TBST buffer. The proteins were then detected with ECL (Shandong Sparkjade Bio‐technology Co., Ltd., #ED0015). The WB antibody rapid stripping buffer (Epizyme, #PS107) was used to strip the primary antibody. The Anti‐Phospho‐OGT (Thr444) antibody was customised by ImmunoWay, as previously reported.^49^ Additional antibody details are provided in the supplementary materials. The protein levels were measured by Image J.

### 2.5 CRISPR‐Cas9‐mediated generation of the NCI‐H1299‐NRF2‐S103A cell line

The NCI‐H1299‐NRF2‐S103A cell line was generated by Cyagen Biosciences (Guangzhou, China). In brief, guide‐RNA target sequence was gRNA1 (5′‐GGAGTAGTTGGCAGATCCACTGG‐3′) and gRNA2 (5′‐ACTCTGTACCTGGGAGTAGTTGG‐3′), and the oligo sequence was TAGATGAAGAGACAGGTGAATTTCTCCCAATTCAGCCAGCCCAGCACATCCAGTCAGAAACTAGTGGATCTGCTAACTACGCCCAGGTACAGAGTACTCAGTTCTTGGGAAAGTTATGGCAGGTTTAAGGAAACACTGAGCAA. This *NFE2L2* gene targeting template was modified by site‐directed mutagenesis to include the S103A mutation (TCC to GCC) and two silent ‘tagging’ changes at the codon for residue T96 (ACC to ACT) and A100 (GCC to GCT). Cas9, gRNA, and donor oligo were co‐transfected into NCI‐H1299 cells by electrotransmission. After single‐cell screening, colonies underwent additional analysis through PCR.

### 2.6 In vivo xenograft mouse study

Female Balb/c‐nude mice aged 4 to 5 weeks were acquired from Beijing Vital River Laboratory Animal Technology Co., Ltd. Subcutaneous inoculation of 1×10^6^ cells were performed on right flanks. Tumour sizes were assessed every three days. Tumour volume = length×width^2^/2. Following a period of 5−6 weeks, the growths were accurately removed, photographed, and their weight measured. More than six mice were used to evaluate each pair of cells. For the cisplatin (MCE, #HY‐17394) treatment, the mice were inoculated with an equal number of NCI‐H1299^NRF2‐WT^ and NCI‐H1299^NRF2‐S103A^ cells. Once the tumour xenografts reached approximately 100 mm^3^, the mice were randomly assigned to three groups for different treatments: PBS, cisplatin at 1.5 mg/kg, and cisplatin at 3.0 mg/kg (administered intraperitoneally once a week for 4 weeks). Every group contained a minimum of 6 mice. During the course of the treatment, the mice's tumour sizes and body weights were assessed at four‐day intervals.

### 2.7 Clinical specimens

Relevant lung cancer tissue specimens and their respective healthy controls were obtained from surgical patients. Samples were gathered exclusively from the Department of Thoracic Surgery, Qingdao Municipal Hospital (Qingdao, China). Follow‐up procedures were described as has been stated.

### 2.8 Protein stability assays

Protein stability was assessed by cycloheximide chase assay, as outlined in the previous study.^50^ Briefly, cells were either transfected or exposed to specified conditions. The medium was supplemented with cycloheximide (CHX, MCE, #HY‐12320). Cells were harvested at specific time points, followed by measuring protein levels through immunoblot analysis.

### 2.9 Statistical analysis

Mean values with standard deviation (*M* ± SD) are reported in this study. To assess normality in each dataset, the Shapiro‐Wilk test was applied. Depending on the fulfilment of test assumptions, either parametric tests (unpaired two‐tailed Student's *t*‐test) or nonparametric tests (Mann–Whitney test) were conducted. Datasets with similar variances were subjected to one‐way ANOVA, followed by post‐hoc tests such as Dunnett's or Tukey's multiple comparisons. For datasets with unequal variances, the Welch's *t*‐test was utilised, along with Brown‐Forsythe analysis of variance and Dunnett's T3 multiple comparisons post hoc tests for sample sizes less than 50. Additionally, two‐way ANOVA with Dunnett's tests was employed to examine protein stability and analyse tumour volume. Statistical analyses were performed using GraphPad Prism 9, with significance levels set at *p < .05, **p < .01, and ***p < .001. The specific tests used for each dataset are detailed in the figure legends.

## 3 RESULTS

### 3.1 O‐GlcNAcylation stabilises and activates NRF2

**FIGURE 1:**
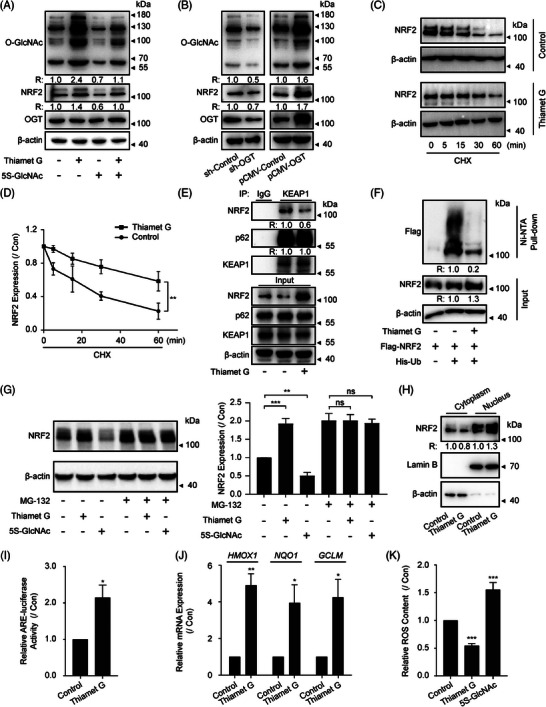
**Global O‐GlcNAcylation regulates the stability and function of NRF2**. (A) NRF2, OGT and global O‐GlcNAcylation expression in NCI‐H1299 cells treated with Thiamet G (2 µM) or/and 5S‐GlcNAc (50 µM) for 2 h was probed by WB. Data represent three independent experiments. (B) The expression of NRF2, OGT, and global O‐GlcNAcylation were determined in NCI‐H1299 cells after OGT KD or overexpression. Data represent three independent experiments. (C and D) NCI‐H1299 cells were pretreated with Thiamet G (2 µM) for 2 h, treated with 2 µg/mL CHX, and incubated for the indicated durations (data represent three independent experiments, **p  <  .01, using two‐way ANOVA followed by Dunnett's multiple comparisons test). (E) The interaction between NRF2, KEAP1, and p62 was determined by a Co‐IP assay with the treatment of Thiamet G (2 µM) for 2 h. Data represent three independent experiments. (F) NCI‐H1299 cells were treated with Thiamet G (2 µM) for 2 h and subjected to an in vivo ubiquitination assay to detect ubiquitin‐conjugated NRF2 proteins. Data represent two independent experiments. (G) NRF2 protein levels were determined by WB in indicated treatment for 2 h (Thiamet G, 2 µM; 5S‐GlcNAc, 50 µM; MG132, 10 µM) (data represent three independent experiments, **p  <  .01; ***p  <  .001; ns, not significant, using one‐way ANOVA followed by Tukey's multiple comparisons test). (H) The distribution of NRF2 was determined by nuclear and cytoplasmic fraction in NCI‐H1299 cells treated with Thiamet G (2 µM) for 2 h. Lysates were separated into cytosolic and nuclear fractions for immunoblotting by indicated antibodies. Lamin B and β‐actin are loading controls for nuclear and cytosolic fractions, respectively. Data represent three independent experiments. (I) NCI‐H1299 cells were transfected with the ARE luciferase reporter and at 24 h post‐transfection, the cells were treated with Thiamet G (2 µM) for 2 h and then analysed for luciferase activity (data represent three independent experiments, *p  <  .05, using unpaired two‐tailed Student's *t*‐test). (J) The expression of *HMOX1*, *NQO1* and *GCLM* were measured by real‐time RT‐PCR in NCI‐H1299 cells with a similar treatment in panel I (data represent three independent experiments, *p  <  .05; **p  <  .01, using unpaired two‐tailed Student's *t*‐test). (K) ROS levels, as indicated by DCFH‐DA fluorescence, were measured by microplate reader in NCI‐H1299 cells, which were treated with Thiamet G (2 µM) or 5S‐GlcNAc (50 µM) for 2 h (data represent four independent experiments, ***p  <  .001, using one‐way ANOVA followed by Dunnett's multiple comparisons test). The relative intensities of proteins in immunoblotting were determined by normalising the intensities of corresponding proteins to the intensities of β‐actin.

Regulation of global O‐GlcNAcylation was conducted to explore the potential role of O‐GlcNAcylation in NRF2. Thiamet G, a validated OGA inhibitor,^51^ and 5S‐GlcNAc, an OGT inhibitor^52^ were employed to modulate the overall cellular O‐GlcNAcylation by blocking OGA and OGT, respectively (Figure 1A). We found that Thiamet G and 5S‐GlcNAc elevated and decreased NRF2 protein levels, respectively (Figure 1A). Moreover, we observed a time‐dependent elevation of NRF2 during Thiamet G treatment and NRF2 protein significantly increased at 2 h after the treatment and then decreased (Figure S1A). Cell lines with OGT knockdown or overexpression were used to validate the impact of O‐GlcNAcylation on NRF2 and eliminate any unintended consequences of small molecule inhibitors. Our discovery revealed that increased OGT expression contributes to a prominent rise in overall O‐GlcNAcylation as well as NRF2 protein, and the depletion of OGT showed a contrasting effect which was consistent with Figure 1A (Figure 1B). The collected data indicates that the global O‐GlcNAcylation regulates the levels of NRF2 protein.

It is well known that the upregulation of protein can be attributed to increased mRNA levels and/or reduced protein degradation.^53^ Thiamet G robustly enhanced NRF2 protein expression instead of mRNA expression (Figures 1A and S1B), indicating a post‐transcriptional mechanism, possibly through the regulation of protein stability. To investigate this, we used cycloheximide (CHX) to block translation elongation and evaluate the stability of NRF2 protein. As expected, the levels of NRF2 protein in untreated samples decreased progressively over time following CHX treatment (Figure 1C and D), and Thiamet G pretreatment increased NRF2 stability in both NCI‐H1299 and HEK293T cells (Figures 1C and D and S1C). However, Thiamet G pretreatment in A549 cells failed to prevent the degradation of NRF2 (Figure S1D). A significant distinction between NCl‐H1299 and A549 cells is the presence of a mutant KEAP1. We hypothesised that Thiamet G could enhance NRF2 stability by controlling KEAP1. In support of this, Thiamet G significantly hindered the interaction between NRF2 and KEAP1 (Figure 1E) and reduced polyubiquitinated NRF2 levels (Figure 1F). Of note, the ubiquitin‐proteasome pathway is the crucial pathways for protein degradation, and significantly contributes to protein stability.^54^ The general proteasome inhibitor MG132 raised NRF2 protein levels and eliminated 5S‐GlcNAc's impact on NRF2 protein levels (Figure 1G), suggesting that O‐GlcNAcylation may inhibit proteasome‐mediated NRF2 degradation. Relevant studies have shown that a classical receptor of autophagy, p62/ SQSTM1 also promoted NRF2 activation via the noncanonical pathway, that is, promoting KEAP1 degradation by binding to KEAP1.^22^, ^23^ However, the activation of NRF2 by O‐GlcNAcylation is likely p62 independent. In this research, there were no significant alterations observed in the levels of p62, LC3 and KEAP1 following stimulation with Thiamet G and 5S‐GlcNAc (Figure S1E). Despite the significant inhibition of NRF2 binding to KEAP1 by Thiamet G, there was no impact on the interaction between KEAP1 and p62 (Figure 1E), indicating stabilisation and activation of NRF2 by O‐GlcNAcylation did not depend on p62‐mediated recruitment of KEAP1 into the autophagosome. Meanwhile, lysosome inhibitor chloroquine had no notable impact on NRF2 protein level induced by Thiamet G and 5S‐GlcNAc (Figure S1F). These findings suggest that O‐GlcNAcylation plays a role in the stabilisation of the NRF2 protein by directly impeding KEAP1‐mediated degradation.

As a pivotal transcription factor, NRF2 has the capability to translocate into the nucleus and form a complex with the antioxidant response element (ARE), thereby instigating the transcription of downstream antioxidant genes, consequently modulating the level of ROS. To validate the impact of O‐GlcNAcylation on NRF2 function, we detected NRF2 nuclear translocation. Thiamet G was found to induce the endo‐nuclear accumulation and alter the cellular distribution of NRF2 (Figure 1H). In line with this, the transcriptional activity of ARE (Figure 1I), and target gene expression (*HMOX1*, *NQO1* and *GCLM*) were upregulated by Thiamet G (Figure 1J). In addition, intracellular ROS were downregulated by higher global O‐GlcNAcylation (Figure 1K). Together, these findings show that O‐GlcNAcylation helps maintain the stability of NRF2 protein by blocking protein degradation through ubiquitin‐proteasome pathway, ultimately boosting its activity.

### 3.2 OGT O‐GlcNAcylates NRF2 at the Ser103 residue

**FIGURE 2:**
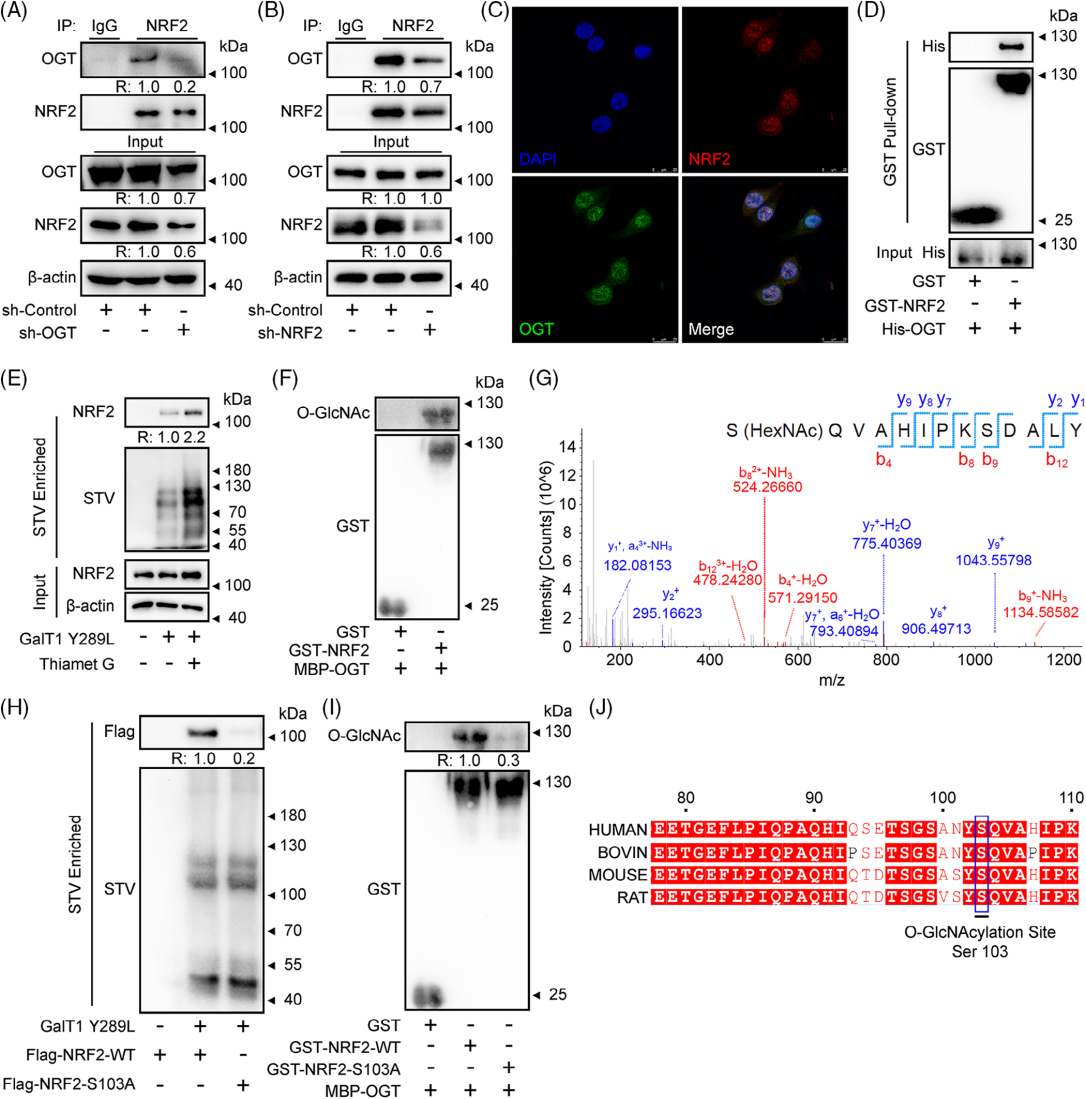
**NRF2 is directly bound to OGT and O‐GlcNAcylated at Ser103**. (A and B) The interaction between NRF2 and OGT was determined by a Co‐IP assay. NCI‐H1299 cells were extracted and immunoprecipitated with control IgG or anti‐NRF2. (C) Co‐localisation of endogenous OGT (green) and NRF2 (red) was determined by immunofluorescence in NCI‐H1299 cells. Scale bars = 20 µm. (D) Direct interaction of OGT with NRF2 in an in vitro GST pull‐down assay. (E) NCI‐H1299 cells, which were pretreated with 2 µM Thiamet G for 2 h, were lysed and labelled by chemoenzymatic labelling. Then the biotinylated proteins were enriched and probed with indicated antibodies and STV‐HRP. (F) GST or GST‐NRF2 was co‐expressed with MBP‐OGT in *E. coli* BL21(DE3) cells. After purification, the protein was immunoblotted with antibodies against O‐GlcNAc (RL2). (G) LC‐MS/MS detected the O‐GlcNAcylated site of NRF2 (Ser103). (H) Flag‐tagged NRF2^WT^ or NRF2^S103A^ vector was transfected into NCI‐H1299 cells. The cells were lysed after treatment with 2 µM Thiamet G for 2 h, followed by chemoenzymatic labelling and WB. (I) NRF2^WT^ or NRF2^S103A^ was co‐expressed with MBP‐OGT in *E. coli* BL21(DE3) cells. After purification, the protein was immunoblotted with antibodies against O‐GlcNAc (RL2). (J) Sequence alignment of NRF2 in different species. Data represent three independent experiments. The relative intensities of proteins in immunoblotting were determined by normalising the intensities of corresponding proteins to the intensities of β‐actin.

The potential link between O‐GlcNAcylation and NRF2 was investigated by detecting the interaction of OGT with NRF2, as the only enzyme that adds N‐acetylglucosamine to the substrate for O‐GlcNAcylation. Co‐immunoprecipitation (Co‐IP) results showed that there was an interaction between NRF2 and OGT (Figure 2A and B). Then, the immunofluorescence results showed that NRF2 and OGT showed spatial co‐localisation (Figure 2C). To investigate whether the OGT‐NRF2 interaction is direct or indirect, the recombinant GST‐NRF2 and His‐OGT were purified and GST pull‐down assays were performed. We observed a direct OGT‐NRF2 interaction (Figure 2D). These data suggest that OGT interacts with NRF2 in both living organisms and laboratory settings, indicating that NRF2 could potentially be a new substrate for OGT.

Next, we used an enzymatic labelling method to detect the O‐GlcNAcylation of NRF2.^55^, ^56^ GalNAz was added directly to the O‐GlcNAc using GalT1 Y289L, followed by biotin click chemistry, and biotinylated proteins were enriched by STV‐conjugated magnetic beads. The findings indicated that NRF2 proteins were able to be detected from the biotinylated proteins which should be O‐GlcNAcylated (Figure 2E). Moreover, the in vitro O‐GlcNAcylation assay revealed that the purified NRF2 could be susceptible to O‐GlcNAcylation by the purified OGT protein (Figure 2F). Collectively, these findings indicate that NRF2 undergoes O‐GlcNAcylation mediated by OGT.

In order to identify the potential O‐GlcNAcylation sites on NRF2, both NRF2 and OGT were co‐expressed in *E. coli* following previous methods,^57^ and the purified NRF2 were analysed by SDS‐PAGE. Subsequent staining NRF2 strips with coomassie brilliant blue was followed by a mass spectrometry (MS) assay, which revealed the 4 potential O‐GlcNAcylation sites (Ser103, Ser111, Ser310 and Thr350) (Figures 2G and S2A‐C). To validate the O‐GlcNAcylation sites obtained from MS assay and identify the primary O‐GlcNAcylation site, NCI‐H1299 cells were transfected with Flag‐tagged NRF2^WT^, NRF2^S103A^, NRF2^S111A^, NRF2^S310A^, or NRF2^T350A^ and their O‐GlcNAcylation levels were measured (Figure S2D). Interestingly, the S103A mutation was the only one that resulted in a significant reduction in O‐GlcNAcylation of NRF2 (Figures 2H and I and S2D), and doesn't affect the interaction of NRF2 and OGT (Figure S2E), indicating that Ser103 is the primary site for NRF2 O‐GlcNAcylation. Moreover, Ser103 is highly conserved within species (Figure 2J).

### 3.3 O‐GlcNAcylation at the Ser103 site stabilises and activates NRF2 via suppressing its binding to KEAP1

**FIGURE 3:**
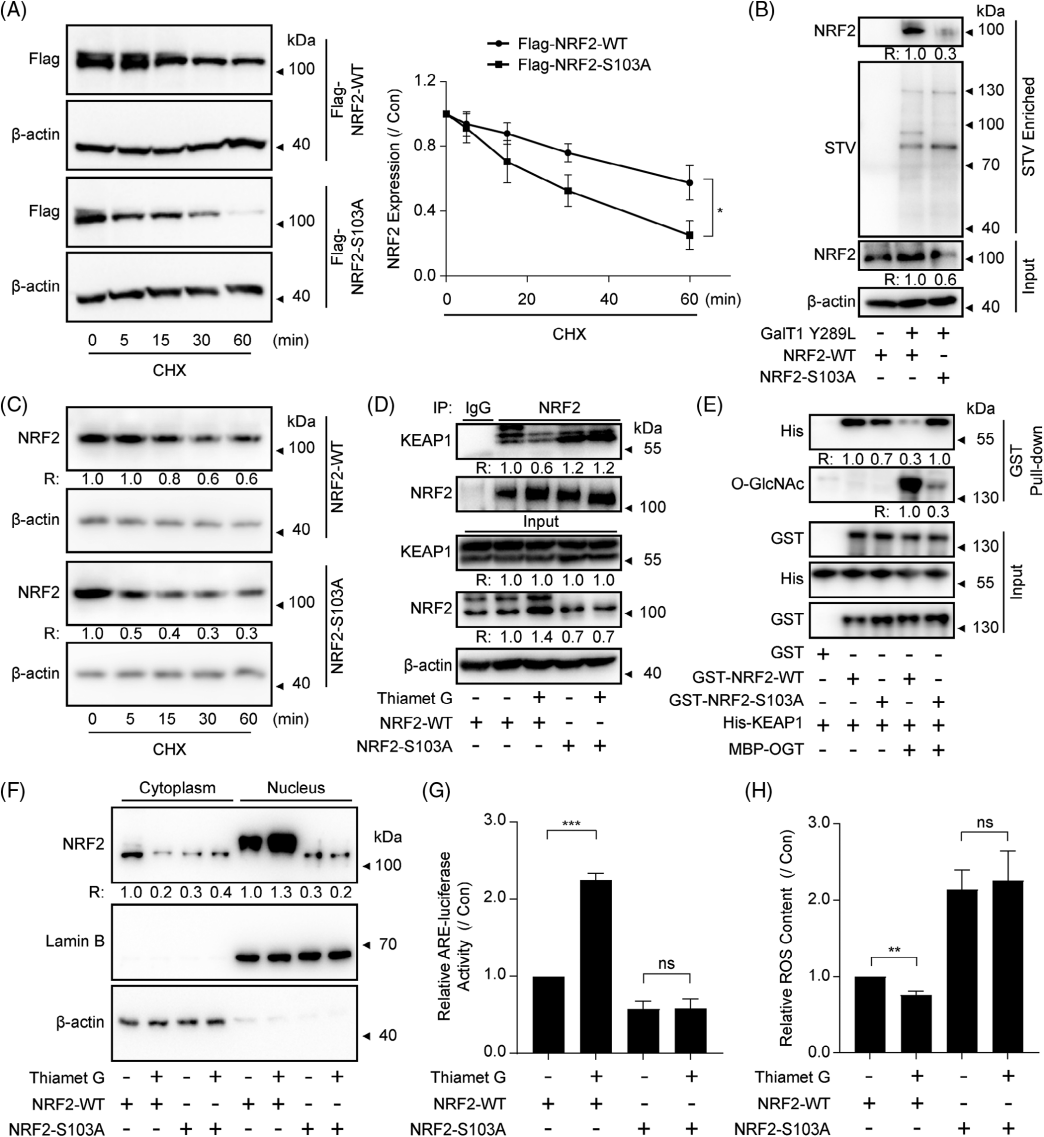
**O‐GlcNAcylation at Ser103 stabilises and activates NRF2 by inhibiting its binding to KEAP1**. (A) NCI‐H1299 cells were translated Flag‐tagged NRF2^WT^ or NRF2^S103A^ and then pretreated with 2 µM Thiamet G for 2 h. The stability of NRF2 was determined by WB and quantification of the Flag band intensity by ImageJ in the presence of CHX for the indicated periods (data represent three independent experiments, *p  <  .05, using two‐way ANOVA followed by Dunnett's multiple comparisons test). (B) The O‐GlcNAcylation of NRF2 was detected by chemoenzymatic labelling in NCI‐H1299^NRF2‐WT^ and NCI‐H1299^NRF2‐S103A^ cells. Data represent two independent experiments. (C) The stability of NRF2 was determined by WB in NCI‐H1299^NRF2‐WT^ and NCI‐H1299^NRF2‐S103A^ cells. Data represent three independent experiments. (D) The interaction between KEAP1 and NRF2 in NCI‐H1299^NRF2‐WT^ and NCI‐H1299^NRF2‐S103A^ cells with or without treated with 2 µM Thiamet G for 2 h. Data represent three independent experiments. (E) Either GST‐tagged NRF2^WT^ or NRF2^S103A^ was co‐expressed with or without MBP‐tagged OGT in *E. coli* BL21(DE3). After purification, an in vitro GST pull‐down assay was performed to detect the KEAP1 bound to NRF2 with or without O‐GlcNAcylation. NRF2 was immunoprecipitated with an anti‐GST antibody, O‐GlcNAcylation of NRF2 was detected by RL2, and then KEAP1 bound to NRF2 was detected by His antibody. Data represent two independent experiments. (F) Relative amounts of NRF2 in the nuclear or cytoplasmic fractions were determined by a cell fraction assay after treatment with 2 µM Thiamet G for 2 h. Data represent two independent experiments. (G) ARE‐luc activity was determined by luciferase reporter assay after treatment with 2 µM Thiamet G for 2 h in NCI‐H1299^NRF2‐WT^ and NCI‐H1299^NRF2‐S103A^ cells (data represent three independent experiments, ***p  <  .001; ns, not significant, using one‐way ANOVA followed by Tukey's multiple comparisons test). (H) ROS levels, as indicated by DCFH‐DA fluorescence, were measured in NCI‐H1299^NRF2‐WT^ and NCI‐H1299^NRF2‐S103A^ cells with the similar treatment in panel G (data represent five independent experiments, **p  <  .01; ns, not significant, using one‐way ANOVA followed by Tukey's multiple comparisons test). The relative intensities of proteins in immunoblotting were determined by normalising the intensities of corresponding proteins to the intensities of β‐actin.

To explore potential influence of O‐GlcNAcylation on NRF2 stability at the Ser103 site, we detected the stability of NRF2 in NCI‐H1299 or HEK293T cells transfected with the site‐mutant NRF2. A more pronounced degradation of NRF2 was noticed in NCI‐H1299 or HEK293T cells transfected with Flag‐tagged NRF2^S103A^, as opposed to NRF2^S111A^, NRF2^S310A^ or NRF2^T350A^. In comparison to transfection with Flag‐tagged NRF2^WT^, this degradation was particularly evident (Figures 3A and S3A‐E), suggesting the O‐GlcNAcylation at Ser103 of NRF2 is critical for its stabilisation. To validate the role of the Ser103 site, we used CRISPR‐Cas9 to construct *NFE2L2* site mutation in NCI‐H1299 cell lines (307T → G, S103A). The *NFE2L2* site mutation was confirmed by sequencing (Figure S3F) and the O‐GlcNAcylation of NRF2 was almost abolished in NCI‐H1299^NRF2‐S103A^ cells (Figure 3B). In addition, more striking NRF2 degradation was observed in NCI‐H1299^NRF2‐S103A^ cells (Figure 3C). Moreover, we performed Co‐IP assay in NCI‐H1299^NRF2‐WT^ and NCI‐H1299^NRF2‐S103A^ cells. The results showed that Thiamet G markedly inhibited the binding of KEAP1 to NRF2^WT^ but not NRF2^S103A^ (Figure 3D). To investigate whether O‐GlcNAcylation of NRF2 hinders its interaction with KEAP1 independent of other protein partners, we co‐expressed GST‐tagged NRF2^WT^ or NRF2^S103A^ with/without OGT in *E. coli* and purified the O‐GlcNAcylated/naked recombinant NRF2. GST‐pulldown assays indicated that both NRF2^WT^ and NRF2^S103A^ could bind KEAP1 without co‐expressing with OGT while co‐expressing with OGT remarkably decreased the binding of KEAP1 with NRF2^WT^ rather than NRF2^S103A^ (Figure 3E). Taken together, these findings illustrated that the NRF2's O‐GlcNAcylation at Ser103 hinders NRF2 interaction with KEAP1.

Then whether NRF2 O‐GlcNAcylation at Ser103 can regulate its function was investigated. It is noteworthy that Thiamet G treatment induced more nuclear translocation of NRF2 in NCl‐H1299^NRF2‐WT^ cells rather than in NCl‐H1299^NRF2‐S103A^ cells (Figure 3F). In line with this, Thiamet G treatment caused increased NRF2 transcription activity and decreased ROS levels in NCl‐H1299^NRF2‐WT^ cells, which was abolished in NCl‐H1299^NRF2‐S103A^ cells (Figure 3G and H). The above findings suggest that Ser103 is a pivotal O‐GlcNAcylation site for NRF2. Furthermore, the protein stability, nuclear translocation, transcription activity, and anti‐oxidation functions of NRF2 was controlled by the specific site.

### 3.4 H_2_O_2_ increases the NRF2 O‐GlcNAcylation through AMPK/OGT pathway

**FIGURE 4:**
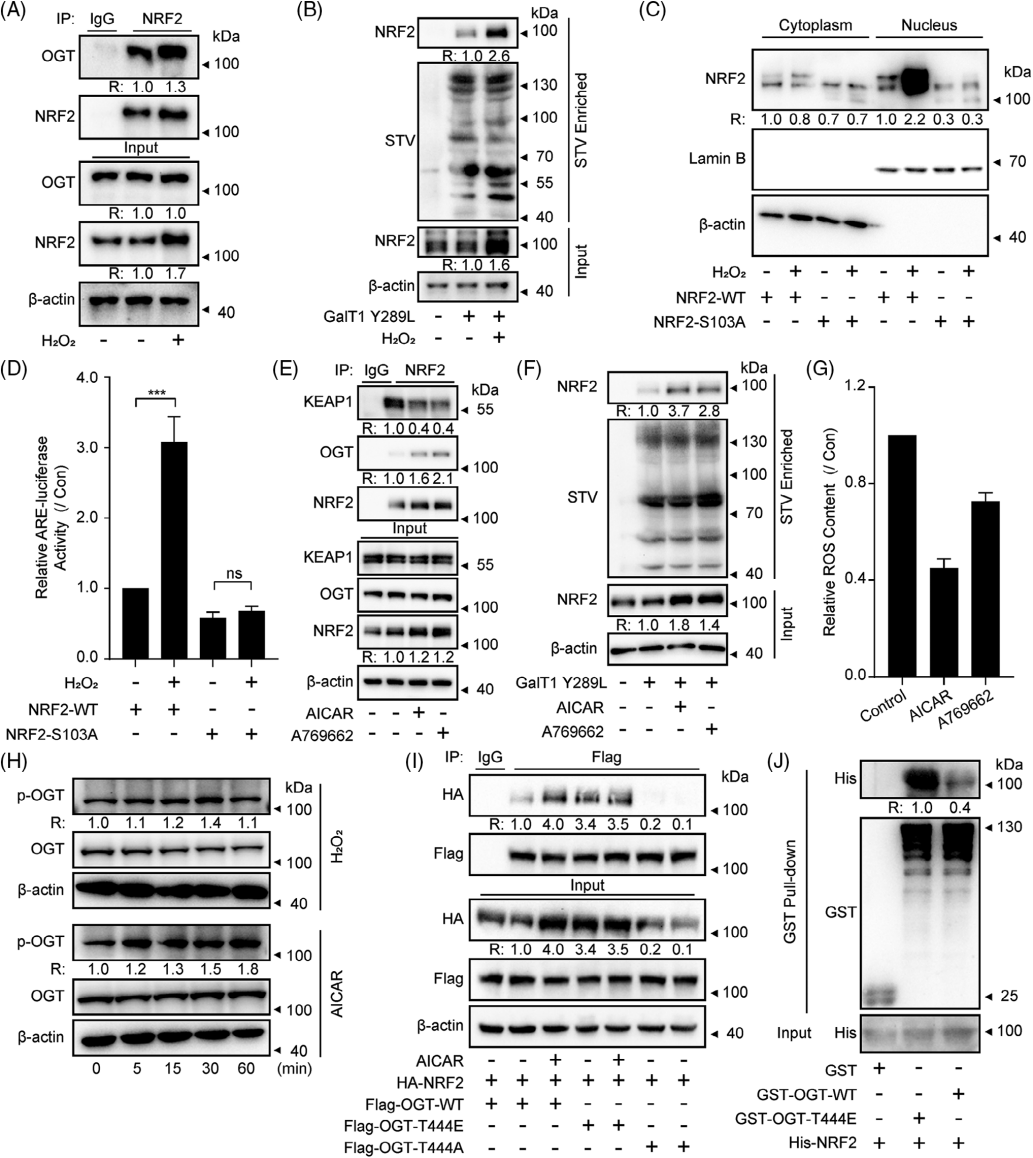
**H_2_O_2_ increases NRF2 O‐GlcNAcylation through AMPK/OGT axis**. (A) Interactions between OGT and NRF2 were determined by Co‐IP assay using NCI‐H1299 cells treated with H_2_O_2_ (50 µM) for 45 min. Data represent three independent experiments. (B) NCI‐H1299 cells were stimulated with H_2_O_2_ (50 µM) for 45 min and O‐GlcNAcylation of NRF2 was detected by chemoenzymatic labelling and WB analysis. Data represent three independent experiments. (C) Relative amounts of NRF2 in the nuclear or cytoplasmic fractions were determined by a cell fraction assay after treatment with H_2_O_2_ (50 µM) for 45 min. Data represent three independent experiments. (D) ARE‐luc activity was determined by luciferase reporter assay in NCI‐H1299^NRF2‐WT^ and NCI‐H1299^NRF2‐S103A^ cells with a similar treatment in panel C (data represent three independent experiments, ***p  <  .001, ns, not significant, using one‐way ANOVA followed by Tukey's multiple comparisons test). (E) The interactions between NRF2 and OGT or KEAP1 were determined by Co‐IP assay in NCI‐H1299 cells treated with AICAR (1 mM) or A‐769662 (50 µM) for 1 h. Data represent three independent experiments. (F) O‐GlcNAcylation of NRF2 was detected by chemoenzymatic labelling and WB analysis in NCI‐H1299 cells treated with AICAR (1 mM) or A‐769662 (50 µM) for 1 h. Data represent three independent experiments. (G) ROS levels, as indicated by DCFH‐DA fluorescence, were measured by microplate reader in NCI‐H1299 cells, which were treated with AICAR (1 mM) or A‐769662 (50 µM) for 1 h (data represent three independent experiments, ***p  <  .001, using one‐way ANOVA followed by Dunnett's multiple comparisons test). (H) Representative images of p‐OGT (Thr444) and OGT proteins determined by WB in the presence of H_2_O_2_ (50 µM) or AICAR (1 mM) for the indicated periods. Data represent three independent experiments. (I) The interaction between OGT and NRF2 was determined by Co‐IP assay after translating Flag‐tagged OGT^WT^, OGT^T444E,^ or OGT^T444A^ in NCI‐H1299 cells. Data represent two independent experiments. (J) Direct interaction of NRF2 with OGT^WT^ and OGT^T444E^ in an in vitro GST pull‐down assay. Data represent two independent experiments. The relative intensities of proteins in immunoblotting were determined by normalising the intensities of corresponding proteins to the intensities of β‐actin.

Recently, studies have showed that cells activate NRF2 in reply to oxidative stress as a defensive mechanism.^58^ The prior mentioned results indicated NRF2 O‐GlcNAcylation increases its protein stability and facilitates NRF2 translocation to the nucleus. This suggests that oxidative stress potentially activates NRF2 through the regulation of NRF2's O‐GlcNAcylation. To investigate this assumption, we detected the impact of H_2_O_2_ on NRF2. In agreement with previous reports,^59^, ^60^ H_2_O_2_ disrupted NRF2 binding to KEAP1, leading to increased nuclear accumulation of NRF2, thereby activating ARE‐luciferase transcription (Figure S4A‐C). Moreover, H_2_O_2_ induced the binding of OGT to NRF2 and increased NRF2 O‐GlcNAcylation in NCI‐H1299 cells (Figure 4A and B). In addition, H_2_O_2_ stimulation induced nuclear translocation and ARE activation in NCl‐H1299^NRF2‐WT^ cells rather than in NCl‐H1299^NRF2‐S103A^ cells (Figure 4C and D). These data together suggest that H_2_O_2_ enhanced NRF2 stability and activity through O‐GlcNAcylation of NRF2 at Ser103.

Subsequently, we investigated the mechanisms underlying the O‐GlcNAcylation of NRF2 induced by H_2_O_2_. Considering both AMPK and NRF2 are oxidative stress sensors, we speculated that H_2_O_2_ might regulate NRF2 O‐GlcNAcylation through AMPK. Indeed, the parallel increases in NRF2, phosphorylation of AMPK (Thr172), and its well‐known substrate acetyl coenzyme A carboxylase (ACC)’s phosphorylation were together detected after H_2_O_2_ stimulation (Figure S4D). To verify the impact of AMPK on NRF2 expression, AMPK activators AICAR and A‐769662 were used. The results demonstrated that both AICAR and A‐769662 significantly upregulated the expression of NRF2 (Figure S4E). Furthermore, AICAR and A‐769662 significantly disrupted the interaction between NRF2 and KEAP1 and increased the binding of OGT to NRF2 in NCI‐H1299 cells (Figure 4E). In parallel, increased NRF2 O‐GlcNAcylation and decreased ROS in AICAR‐ or A‐769662‐treated cells were observed (Figure 4F and G). These findings collectively suggest that AMPK regulates the O‐GlcNAcylation of NRF2, thereby affecting its function.

As an AMP‐dependent protein kinase, AMPK regulates cellular functions by phosphorylating various proteins. It has been reported that AMPK can phosphorylate OGT at the Thr444 site, thereby regulating its nuclear localisation and substrate selectivity.^49^ Given that AMPK regulated NRF2 O‐GlcNAcylation, it is speculated that AMPK regulated the interaction between NRF2 and OGT via the OGT phosphorylation. Interestingly, the phosphorylation of OGT (Thr444) exhibited a time‐dependent upregulation under H_2_O_2_ or AICAR stimulation (Figure 4H). In order to validate the significance of OGT phosphorylation, we prevented or mimicked OGT phosphorylation at the Thr444 site by the T444A or T444E mutation, respectively. The Co‐IP results showed that the T444A mutation weakened the interaction between OGT and NRF2, while the T444E mutation enhanced the binding of NRF2 with OGT (Figure 4I). In line with the aforementioned findings, GST‐pulldown assays demonstrated that the GST‐OGT^T444E^ exhibited stronger binding to His‐NRF2 in vitro (Figure 4J), suggesting that OGT phosphorylation at the Thr444 by AMPK is of great significance in modulating the interaction between OGT and NRF2. These above results illustrate that H_2_O_2_ increases NRF2 O‐GlcNAcylation by activating the AMPK/OGT axis.

### 3.5 O‐GlcNAcylation of NRF2 at Ser103 enhances malignancy of lung cancer cells in vitro and in vivo

**FIGURE 5:**
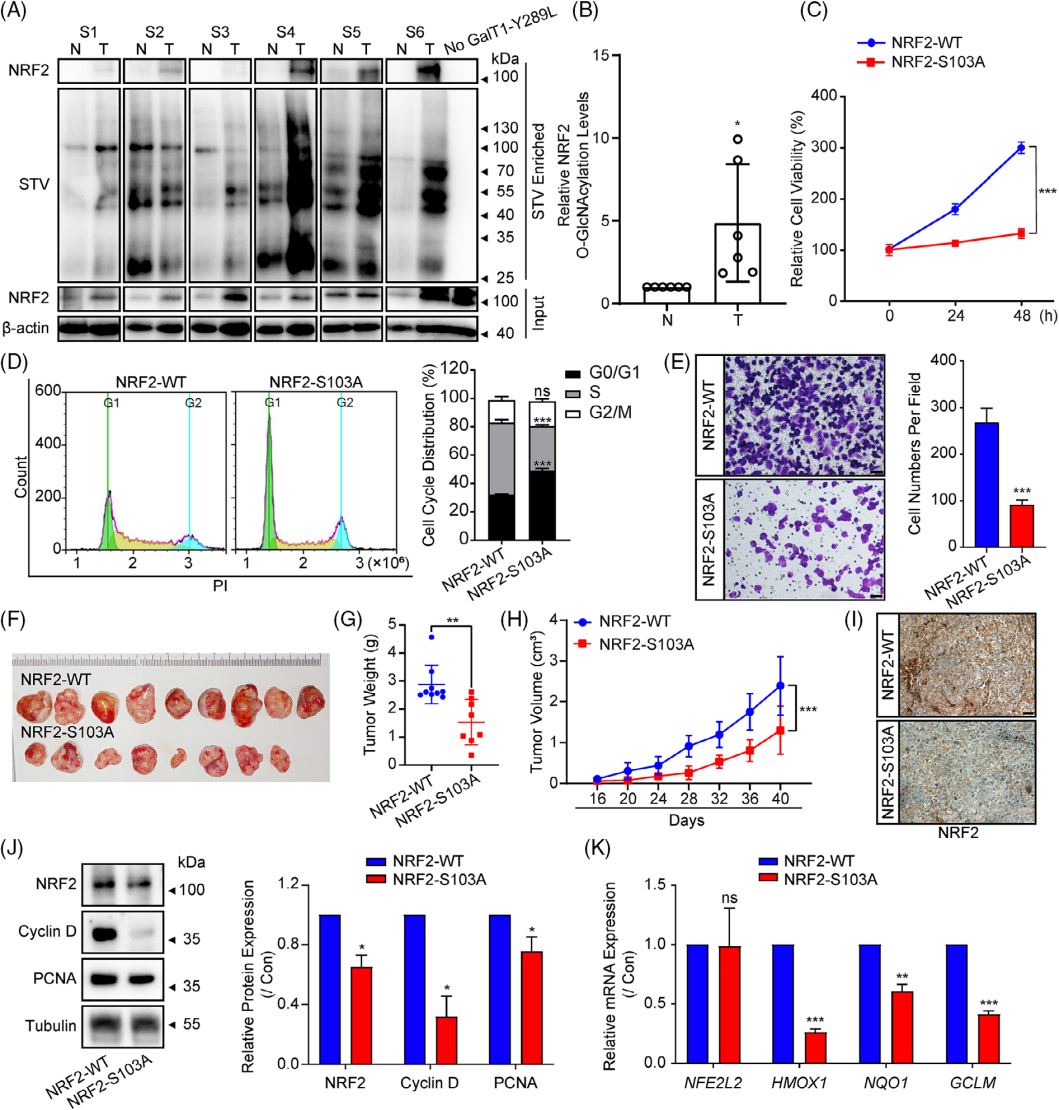
**NRF2 O‐GlcNAcylation at Ser103 enhances lung cancer cell growth both in vitro and in vivo**. (A) O‐GlcNAcylation of NRF2 was detected by chemoenzymatic labelling and WB analysis in 6 human lung cancers (T) and paired adjacent normal controls (N). (B) Quantification of NRF2 O‐GlcNAcylation in human lung cancer samples. (*n* = 6, *p  <  .05, using paired two‐tailed Student's *t*‐test). (C) The proliferation of NCI‐H1299^NRF2‐WT^ and NCI‐H1299^NRF2‐S103A^ cells were evaluated by MTT assay (data represent six independent experiments, ***p  <  .001, using two‐way ANOVA followed by Dunnett's multiple comparisons test). (D) Flow cytometry was used to determine the distribution of cells in different phases of the cell cycle in NCI‐H1299^NRF2‐WT^ and NCI‐H1299^NRF2‐S103A^ cells (data represent three independent experiments, ***p  <  .001; ns, not significant, using unpaired two‐tailed Student's *t*‐test). (E) Cell migration of NCI‐H1299^NRF2‐WT^ and NCI‐H1299^NRF2‐S103A^ cells was detected by the transwell assay (data represent four independent experiments, ***p  <  .001, using unpaired two‐tailed Student's *t*‐test). (F) The xenografts were harvested and the morphology of tumour xenografts were photographed. (G) The tumour weight from each mouse was measured (**p  <  .01, using Mann–Whitney Student's *t*‐test, NCI‐H1299^NRF2‐WT^ group, *n* = 9; NCI‐H1299^NRF2‐S103A^ group, *n* = 8). (H) The tumour volume of each mouse from different groups was measured (***p  <  .001, using two‐way ANOVA followed by Dunnett's multiple comparisons test, NCI‐H1299^NRF2‐WT^ group, *n* = 9; NCI‐H1299^NRF2‐S103A^ group, *n* = 8). (I) The expression of NRF2 was detected in samples derived from NCI‐H1299 xenografts using immunohistochemistry. Scale bars = 50 µm. Data represent four independent experiments. (J, K) The expression levels of NRF2, Cyclin D, and PCNA were determined by WB (J), and NRF2 target genes were measured by real‐time RT‐PCR (K) in samples derived from NCI‐H1299 xenografts (data represent three independent experiments, *p  <  .05; **p  <  .01; ***p  <  .001; ns, not significant, using unpaired two‐tailed Student's *t*‐test). The relative intensities of proteins in immunoblotting were determined by normalising the intensities of corresponding proteins to the intensities of β‐actin and Tubulin.

To examine pathophysiologic significance of NRF2 and O‐GlcNAcylation in lung carcinoma, we initially assessed the overall levels of O‐GlcNAcylation and NRF2 expression in 6 paired human lung cancer samples. Remarkably, a strong connection was noted between the NRF2 protein levels and overall O‐GlcNAcylation levels (Figures 5A and S5A and B, *r* = .7467, *p* = .0053). Additionally, we identified the O‐GlcNAcylation of NRF2 in lung cancer samples through the enzyme labelling technique, revealing a substantial increase in O‐GlcNAcylation levels of NRF2 within tumour tissues in comparison with adjacent tissues (Figure 5A and B). The GEPIA^61^ analysis of TCGA^62^ also showed that the NRF2 mRNA expression level did not show any statistically significant disparity between tumour and normal tissues (Figure S5C), which means that the regulation of NRF2 may primarily occur through the PTM, which modulate protein stability of NRF2 and subsequently impact its function. The findings indicate the O‐GlcNAcylation of NRF2 may be significantly involved in development of lung cancer. Considering the significant impact of NRF2 on cancer aggressiveness, these investigations indicate that NRF2 O‐GlcNAcylation could play a part in promoting malignant cell actions. To test this hypothesis, we conducted experiments on cell growth, formation of colonies, and migration in NCI‐H1299^NRF2‐WT^ and NCI‐H1299^NRF2‐S103A^ cells. The growth of NCI‐H1299^NRF2‐S103A^ cells was significantly reduced, and cell cycle arrest was notably enhanced compared to NCI‐H1299^NRF2‐WT^ cells (Figure 5C and D). Additionally, the transwell assay showed that NCI‐H1299^NRF2‐S103A^ cells displayed reduced migration in comparison with NCI‐H1299^NRF2‐WT^ cells (Figure 5E). Collectively, our findings suggest that NRF2 O‐GlcNAcylation at Ser103 enhances the aggressive characteristics of lung cancer in vitro. To explore the biological importance of NRF2 O‐GlcNAcylation, we conducted subcutaneous xenograft experiments using NCI‐H1299^NRF2‐WT^ and NCI‐H1299^NRF2‐S103A^ cells. S103A mutation of NRF2 significantly reduced subcutaneous tumour growth, and mice inoculated with NCI‐H1299^NRF2‐S103A^ cells developed smaller tumours compared to those inoculated with NCI‐H1299^NRF2‐WT^ cells at the time of harvesting (Figures 5F‐H and S5D). In parallel, the expression levels of NRF2, Cyclin D, and PCNA was observed to be reduced in xenografts derived from NCI‐H1299^NRF2‐S103A^ cells (Figure 5I and J). Moreover, the mRNA levels of NRF2 targets, but not NRF2 itself, were decreased in xenografts derived from NCI‐H1299^NRF2‐S103A^ cells (Figure 5K). Overall, these findings suggest that the NRF2 O‐GlcNAcylation at Ser103 promotes lung cancer malignancy in vitro and in vivo.

### 3.6 NRF2 O‐GlcNAcylation at Ser103 decreases cellular ROS levels and enhances lung cancer cells' survival during cisplatin treatment

**FIGURE 6:**
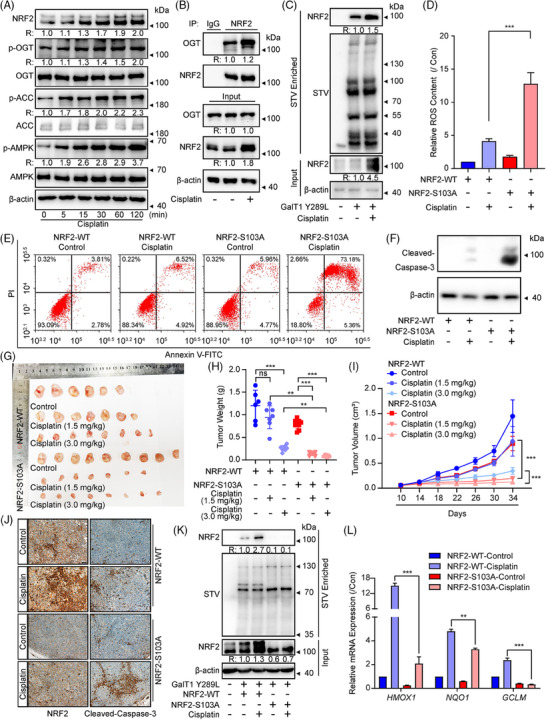
NRF2 O‐GlcNAcylation at Ser103 decreases ROS level and enhances lung cancer cell survival during cisplatin treatment. (A) The expression of p‐OGT, OGT, p‐ACC, p‐AMPK, and NRF2 were measured by WB in NCI‐H1299 cells with cisplatin (10 µM) for the indicated periods. Data represent two independent experiments. (B) Interactions between OGT and NRF2 were determined by Co‐IP assay using NCI‐H1299 cells treated with cisplatin (10  µM) for 2 h. Data represent two independent experiments. (C) NCI‐H1299 cells were stimulated with cisplatin (10 µM) and O‐GlcNAcylation of NRF2 was detected by chemoenzymatic labelling and WB analysis. Data represent three independent experiments. (D) ROS levels were determined in NCI‐H1299^NRF2‐WT^ and NCI‐H1299^NRF2‐S103A^ cells in the presence or absence of cisplatin (10 µM) for 2 h by DCFH‐DA fluorescence (data represent five independent experiments, ***p  <  .001, using one‐way ANOVA followed by Tukey's multiple comparisons test). (E) Apoptosis level, as revealed by Annexin V staining was determined in NCI‐H1299 cells with cisplatin (10 µM) treatment. Data represent two independent experiments. (F) The cleaved and total caspase‐3 were determined by WB in NCI‐H1299^NRF2‐WT^ and NCI‐H1299^NRF2‐S103A^ cells with a similar treatment in panel E. Data represent three independent experiments. (G) The xenografts were harvested and the morphology of xenografts of NCI‐H1299^NRF2‐WT^ and NCI‐H1299^NRF2‐S103A^ cells with or without cisplatin (1.5 mg/kg or 3.0 mg/kg) treatments were photographed. (H) The tumour weight of each nude mouse was measured (**p  <  .01; ***p  <  .001; ns, not significant, using one‐way ANOVA followed by Tukey's multiple comparisons test, *n* ≥ 6 for each pair). (I) The tumour volume of each mouse from different groups was measured (***p  <  .001, using two‐way ANOVA followed by Tukey's multiple comparisons test, *n* ≥ 6 for each pair). (J) The expression of NRF2 and cleaved‐caspase 3 were detected by immunohistochemistry staining in samples derived from NCI‐H1299^NRF2‐WT^ and NCI‐H1299^NRF2‐S103A^ xenografts (cisplatin, 3.0 mg/kg, Scale bars = 50 µm). Data represent four independent experiments. (K) The O‐GlcNAcylation of NRF2 was detected in samples derived from NCI‐H1299^NRF2‐WT^ and NCI‐H1299^NRF2‐S103A^ xenografts (cisplatin, 3.0 mg/kg). Data represent two independent experiments. (L) The expression of *HMOX1*, *NQO1* and* GCLM* were measured by real‐time RT‐PCR in samples derived from NCI‐H1299^NRF2‐WT^ and NCI‐H1299^NRF2‐S103A^ xenografts (data represent three independent experiments, **p  <  .01; ***p  <  .001, using one‐way ANOVA followed by Tukey's multiple comparisons test).

The maintenance of ROS balance is essential for maintaining cells viability, and several anti‐cancer medications have been discovered to induce cell apoptosis by producing ROS, including cisplatin, arsenic trioxide, and so on.^63^, ^64^ Taking into account its antioxidative activity, we investigated whether O‐GlcNAcylation of NRF2 at Ser103 could attenuate the chemosensitivity of cancer cells towards chemotherapeutic drugs like cisplatin. First, we confirmed whether cisplatin affected the O‐GlcNAcylation of NRF2 by activating the AMPK/OGT axis. Indeed, cisplatin significantly elevated the phosphorylation of AMPK and OGT (Figure 6A). Moreover, cisplatin also enhanced the binding of OGT to NRF2 and increased NRF2 O‐GlcNAcylation (Figure 6B and C). Then, NCI‐H1299^NRF2‐WT^ and NCI‐H1299^NRF2‐S103A^ cells were employed to investigate the potential involvement of NRF2 O‐GlcNAcylation how cancer cells react to chemotherapeutic agents. As expected, more ROS accumulation and cell apoptosis were observed after cisplatin treatment in NCI‐H1299^NRF2‐S103A^ cells (Figure 6D‐F). The IC50 of cisplatin in NCI‐H1299^NRF2‐WT^ cells was 31.83 µM and 19.53 µM in NCI‐H1299^NRF2‐S103A^ cells. The data presented herein provide compelling evidence that NRF2 O‐GlcNAcylation serves as a pivotal mechanism underlying the development of resistance to cisplatin‐induced apoptosis. Furthermore, the subcutaneous tumour model findings demonstrates that low‐dose cisplatin (1.5 mg/kg) mildly inhibits tumour growth in NCI‐H1299^NRF2‐WT^ cells but completely retards tumour growth in NCI‐H1299^NRF2‐S103A^ cells, which are line with the in vitro results (Figures 6G‐I and S6A). While high‐dose cisplatin (3.0 mg/kg) significantly impeded tumour growth, the presence of the S103A mutation rendered the xenografts more sensitive to cisplatin treatment (Figures 6G‐I and S6A). Cisplatin slightly induced the expression of proapoptotic makers, cleaved caspase‐3, in xenografts derived from NCI‐H1299^NRF2‐WT^ cells, however, more induction of cleaved caspase‐3 was observed in cisplatin‐treated xenografts derived from NCI‐H1299^NRF2‐S103A^ cells (Figure 6J). Moreover, the O‐GlcNAcylation, the expression of NRF2 and its target genes exhibited dramatically suppression in cisplatin‐treated xenografts derived from NCI‐H1299^NRF2‐S103A^ cells (Figure 6J‐L). Collectively, these results indicate that blocking NRF2 O‐GlcNAcylation may offer potential for enhancing the efficacy of cisplatin chemotherapy in lung cancer.

## 4 DISCUSSION

**FIGURE 7:**
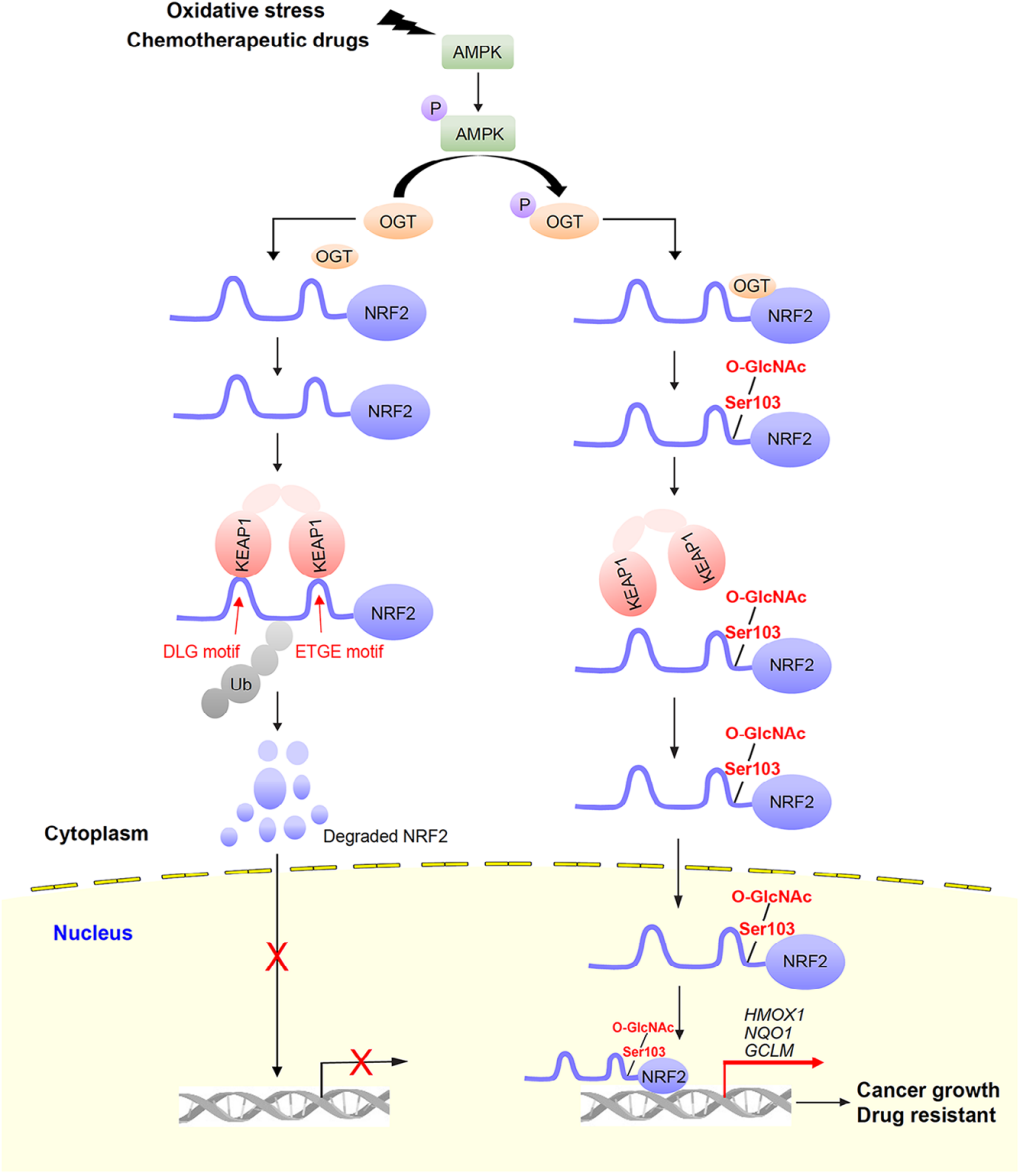
**Working model for regulation of NRF2 activity by O‐GlcNAcylation in cancer cells**. Under normal circumstances, NRF2 is bound to KEAP1, which is a redox‐sensitive E3 ubiquitin ligase substrate adaptor molecule that regulates the ubiquitination and degradation of NRF2 (left). Upon oxidative stress or chemotherapy, AMPK is activated to phosphorylate OGT at the Thr444 site. The activated OGT is conducive to binding with NRF2 to increase the O‐GlcNAcylation of NRF2, which inhibits the binding of KEAP1 to NRF2 and thus promotes its stability, nuclear localisation, and transcription of its target genes, thus driving lung cancer cell proliferation, survival, and drug resistance.

NRF2, a transcription factor that provides protection to cells, has a dual role in cancer. While it shields healthy cells from oxidative harm, it also aids cancer cells in developing resistance to chemotherapy.^63^, ^64^ NRF2 plays a crucial role in repairing cell damage caused by drugs, forming a shield against drugs, and blocking drug entry into cells,^65^ leading to reduced sensitivity of cancer cells with high NRF2 levels to common chemotherapy drugs like 5‐fluorouracil, etoposide and cisplatin.^66^ Here, we prove that OGT‐mediated O‐GlcNAcylation of NRF2 at Ser103 enhances NRF2 protein stability. In addition, we clarify the regulatory mechanism of NRF2 O‐GlcNAcylation: oxidative stress activates AMPK to phosphorylate OGT, which facilitates OGT binding to NRF2, resulting in elevated O‐GlcNAcylation of NRF2 (Figure 7). Despite the detection of elevated levels of O‐GlcNAcylation in various cancer types, our study presents novel evidence demonstrating the physiological O‐GlcNAcylation of NRF2, which is implicated in tumour malignancy and cisplatin resistance. Our study offers fresh perspectives on improving ameliorate chemotherapy resistance through the creation of a unique inhibitor targeting NRF2 O‐GlcNAcylation.

Recently, AMPK became a hot target for tumour therapy for several reasons. Activation of AMPK in cancer cells has been demonstrated to facilitate metabolic reprogramming that are resistant to drugs, such as by augmenting the Warburg effect and promoting mitochondrial biogenesis.^67^ In addition, it has been shown that AMPK activity regulates the ability of cancer stem cells to renew themselves which are usually not affected by chemotherapy.^68^ Additionally, the activation of AMPK is crucial in initiating the autophagy process.^69^ This process has been shown to degrade cellular components to meet nutritional requirements under stressful conditions, thereby effectively regulating chemosensitivity. It is worth noting that in our study, AMPK activators enhanced OGT/NRF2 interaction and NRF2 O‐GlcNAcylation. Hence, inhibiting AMPK could counteract chemotherapy‐induced side effects. This discovery provides a new mechanism for AMPK involvement in chemotherapy resistance, which may further increase the chance and confidence to develop new tumour therapy by focusing on AMPK.

A recent study reported that NRF2 or CUL3 was not O‐GlcNAcylated by using O‐GlcNAc antibody.^70^ We also failed to detect the O‐GlcNAcylation of NRF2 using O‐GlcNAc antibodies (RL2 and CTD110.6) (data not shown). Each O‐GlcNAc antibody may target a unique O‐GlcNAc‐dependent epitope, and antibody‐based methods may lack sensitivity in detecting O‐GlcNAcylation in situations of low abundance or limited sample availability.^71^ Here we confirmed the O‐GlcNAcylation of NRF2 using chemoenzymatic labelling and determined its modification site by mass spectrometry. Given the extensive involvement of O‐GlcNAcylation in regulating numerous proteins, it is important to take into account the impact of other proteins when investigating its effects on the stability of NRF2. For instance, prior investigations have demonstrated that upregulating O‐GlcNAcylation levels leads to the inactivation of the 19S proteasome, thereby exerting regulatory control over protein degradation.^72^ As a crucial regulator, KEAP1 is O‐GlcNAcylated at Ser104 governs the interaction of KEAP1 with CUL3 as well as NRF2 activity under relatively long periods of stimulation.^70^ Nevertheless, our findings demonstrate that the NRF2 S103A mutation significantly diminishes NRF2 stability and eliminates its response to alterations in global cellular O‐GlcNAcylation, thereby suggesting that additional OGT substrates are not required to elucidate these effects within this specific context.

O‐GlcNAcylation and phosphorylation have intricate interactions as they both alter serine or threonine residues.^73^ Here, we have identified the O‐GlcNAcylation at the Ser103 of NRF2 through PhosphoSitePlus (http://www.phosphosite.org). We searched for the presence of multiple phosphorylation modification sites in NRF2 (including sites for high‐throughput prediction and experimental verification) but did not include the Ser103 site. It would be highly intriguing to investigate in future studies the potential phosphorylation of Ser103 and reveal the intricate crosstalk between O‐GlcNAcylation and phosphorylation. Although our current study indicates that O‐GlcNAcylation at Ser103 modulates the interaction between NRF2 and KEAP1 in vivo and in vitro, the precise mechanism by which O‐GlcNAcylation influences this binding remains elusive. A reasonable hypothesis is that the O‐GlcNAcylation of NRF2 at the Ser103 may affect the conformation of NRF2. However, the current eutectic structure of NRF2 and KEAP1 only contains a small segment of NRF2 peptide (about 35 amino ends), so it is difficult to predict whether the O‐GlcNAcylation at Ser103 affects the conformation of NRF2 using molecular dynamics simulates.

Previous study indicated a notable increase in O‐GlcNAcylation and OGT expression in lung cancer tissues when compared to the corresponding adjacent tissues.^45^ In this article, we first present findings indicating a direct relationship between the NRF2 expression and overall O‐GlcNAcylation in lung cancer. Analysis of clinical samples revealed an upregulation of NRF2 O‐GlcNAcylation in human lung tumours. Furthermore, chemotherapy treatment enhanced the NRF2 O‐GlcNAcylation in NCI‐H1299 cells. Our findings not only elucidate the underlying mechanism by which chemotherapy‐induced drug resistance occurs through increased O‐GlcNAcylation of NRF2 but also provide potential therapeutic targets for lung cancer. Furthermore, NRF2 has been considered as a potential target for therapy due to its function in the controlling of critical biological functions. However, obtaining specific inhibitors of NRF2 for cancer therapy without side effects has proven to be difficult.^74^, ^75^ In contrast, it is possible to target protein‐protein interactions involving NRF2. Thus, revealing the protein‐protein interactions that regulate NRF2 will offer a practical and precise approach to inhibiting its activity. Discovering small compounds that exhibit strong efficacy and specificity is essential for creating successful and secure medications. A novel approach to drug design, known as PTM inspired drug design (PTMI‐DD), is emerging as a result of PTM. The primary emphasis of this design is on developing drugs that target endogenous cofactors or substrate binding sites, as well as nearby areas such as covalent inhibitor design, targeted allosteric binding sites, and targeted protein‐protein interfaces.^76^ Our research on NRF2 O‐GlcNAcylation directly leads to the discovery of effective biomarkers and provides a reliable basis for drug design based on PTM.

Recent reports indicate that OGT inhibitors exert anti‐tumour effects through various mechanisms. The application of these inhibitors has shown a significant decrease in neoplastic cells growth. Among them, OSMI‐1, a newly discovered small molecule OGT inhibitor, activates the tumour suppressor ERRFI1 through epigenetic mechanisms, thereby inhibiting breast cancer growth.^77^ Additionally, OGT inhibitors exhibit potential in combination chemotherapy by increasing the sensitivity of prostate cancer cells to docetaxel and enhancing the efficacy of chemotherapy.^78^ Furthermore, OGT inhibitors are increasingly recognised as immune modulation targets, capable of enhancing immune responses within the tumour microenvironment, which offers new strategies for immunotherapy.^79^ The increasing body of research on the impact of O‐GlcNAc modification in tumourigenesis has positioned the utilisation of OGT inhibitors as a pivotal aspect in cancer therapeutics.^80^ However, despite their considerable potential, the development and application of OGT inhibitors face significant challenges. A primary challenge is the low affinity and selectivity of current OGT inhibitors. Among numerous small molecules, only a few effectively target OGT, and these often lack sufficient selectivity, potentially leading to increased side effects and diminished therapeutic efficacy.^81^, ^82^ Pharmacokinetic properties of OGT inhibitors also pose a critical challenge. Current studies indicate that many potential OGT inhibitors exhibit low stability and bioavailability in vivo, limiting their clinical applicability.^83^, ^84^ Additionally, tumour heterogeneity complicates targeted therapy against OGT. Different tumour types exhibit varying degrees of dependence on OGT, suggesting that a single inhibitor may be effective in some tumours but not in others. This underscores the need for personalised treatment research to determine the sensitivity of different tumour types to OGT inhibitors.^85^, ^86^ Moreover, OGT regulates multiple signalling pathways through O‐GlcNAc modification, contributing to its complex role in tumourigenesis and progression. This complexity further complicates the development of effective therapeutic strategies targeting OGT.^80^, ^87^ Therefore, it is imperative for research on O‐GlcNAc modification to prioritise two crucial areas: employing high‐throughput screening techniques to identify inhibitors with enhanced specificity and improved pharmacokinetic properties, and conducting comprehensive investigations into the complex mechanisms that contribute to the influence of O‐GlcNAc modification on tumour development.^87^ Such efforts will facilitate the development of more synergistic treatment approaches, ultimately aiding in the realisation of more precise clinical therapies.^52^, ^86^

Despite the challenge posed by the fluctuating levels of NRF2 and O‐GlcNAcylation in different contexts, it is evident that their involvement is pivotal in disease development. A comprehensive exploration and comprehension of the regulation of NRF2 and O‐GlcNAcylation are likely to hold significant value in devising enhanced therapeutic strategies for diverse diseases in the future.

## AUTHOR CONTRIBUTIONS

Conceptualisation, Yihan Zhang, Yuchao Gu and Wengong Yu Methodology, Yihan Zhang, Changning Sun, Leina Ma, and Guokai Xiao Formal analysis, Yihan Zhang and Changning Sun Investigation, Yihan Zhang, Changning Sun, Leina Ma, and Guokai Xiao Resources, Yihan Zhang and Changning Sun Writing—original draft, Yihan Zhang, Changning Sun, Yuchao Gu and Wengong Yu Writing—review & editing, Yihan Zhang, Changning Sun, Yuchao Gu and Wengong Yu Supervision, Yuchao Gu and Wengong Yu Funding acquisition, Yuchao Gu and Wengong Yu. All authors read and approved the manuscript.

## CONFLICT OF INTEREST STATEMENT

The authors declare no conflicts of interest.

## ETHICS STATEMENT

Animal experiments were conducted in compliance with protocols that were reviewed and approved by the Ethics and Animal Welfare Committee of Ocean University of China (SMP‐2018‐013). Human samples were obtained with patient consent and experiments were approved by the Ethics and Animal Welfare Committee of Ocean University of China (SMP‐2019‐009).

## Supporting information



Supporting Information

## Data Availability

All data included in this study are available upon request by contact with the corresponding author.
