# Supplementary material for: O‐GlcNAcylation promotes malignancy and cisplatin resistance of lung cancer by stabilising NRF2
Source: Clin Transl Med. 2024 Oct 2;14(10):e70037. doi: 10.1002/ctm2.70037 (PMC11447106; doi:10.1002/ctm2.70037)
Supplement: Supplementary file 1 — Supporting Information [file CTM2-14-e70037-s001.docx]

**Supplementary Tables and Figures**

**O-GlcNAcylation promotes malignancy and cisplatin resistance of lung cancer by stabilizing NRF2**

Yihan Zhang^1,2,3,^*, Changning Sun^1,2,3,^*, Leina Ma^1,2,3^, Guokai Xiao^1,2,3^, Yuchao Gu^1,2,3^, and Wengong Yu^1,2,3^

This file includes:

Supplementary materials and methods

Supplementary Table S1

Supplementary Figure S1 to S6

**Supplementary materials (antibodies)**

IgG antibodies, including Normal Mouse IgG (Merck Millipore, #12-371, RRID: AB_145840) and Normal Rabbit IgG (Merck Millipore, #12-370, RRID: AB_145841), were utilized in the Co-IP assay.

The following antibodies were utilized in immunoblotting: Anti-NRF2 (Abcam, #ab62352, RRID: AB_944418), Anti-NRF2 (Proteintech, #16396-1-AP, RRID: AB_2782956), Anti-β-Tubulin (Sigma-Aldrich, #T8328, RRID: AB_1844090), Anti-Flag (Sigma-Aldrich, #F1804, RRID: AB_262044), Anti-HA (Sigma-Aldrich, #H3663, RRID: AB_262051), Anti-HA (Cell Signaling technology, #3724S, RRID: AB_1549585), Anti-His (Sigma-Aldrich, #H1029, RRID: AB_260015), Anti-OGT (Sigma-Aldrich, #O6264, RRID: AB_532313), Anti-OGT (Abcam, #ab96718, RRID: AB_10680015), Anti-OGA (Abcam, #ab124807, RRID: AB_10971848), Anti-O-GlcNAc (RL2) (Abcam, #ab2739 , RRID: AB_303264), Anti-AMPK (Cell Signaling technology, #2532S, RRID: AB_330331), Anti-Phospho-AMPK (Thr172) (Cell Signaling technology, #2531S, RRID: AB_330330), Anti-KEAP1 (D6B12) (Cell Signaling technology, #8047S, RRID: AB_10860776), Anti-KEAP1 (Proteintech, #10503-2-AP, RRID: AB_2132625), Anti-KEAP1 (Proteintech, #60027-1-Ig, RRID: AB_2132623), Anti-β-Actin (Cell Signaling technology, #4967S, RRID: AB_330288), Anti-Cleaved Caspase 3 (Cell Signaling technology, #9664S, RRID: AB_2070042), Anti-Phospho-Acetyl-CoA Carboxylase (Ser79) (Cell Signaling technology, #11818S, RRID: AB_2687505), Anti-Acetyl-CoA Carboxylase (Proteintech, #67373-1-Ig, RRID: AB_2882621), Anti-Cyclin D (Cell Signaling technology, #55506S, RRID: AB_2827374), Anti-PCNA (Cell Signaling technology, #13110S, RRID: AB_2636979), Anti-Lamin B (Proteintech, #12987-1-AP, RRID: AB_2136290), Anti-GST (Abcam, #ab92, RRID: AB_307067), Goat anti-Rabbit IgG (H&L) HRP conjugate (Merck Milipore, #AP156P, RRID: AB_91699), Peroxidase AffiniPure Goat Anti-Mouse IgG (H&L) (Jackson, #115-035-003, RRID: AB_10015289), Streptavidin-HRP (Invitrogen, #434323, RRID: AB_2619743). The Anti-Phospho-OGT (Thr444) antibody was customized by ImmunoWay

The following antibodies were utilized in immunofluorescence microscopy: Alexa Fluor 488-conjugated goat anti-mouse IgG (H&L) (Invitrogen, #A11029, RRID: AB_2534088), Alexa Fluor 594-conjugated goat anti-rabbit IgG (H&L) (Invitrogen, #A11012, RRID: AB_2534079), Alexa Fluor 488-conjugated goat anti-rabbit IgG (H&L) (Abcam, #ab150077, RRID: AB_2630356), Alexa Fluor 594-conjugated goat anti-mouse IgG (H&L) (Abcam, #ab150116, RRID: AB_2650601).

**Supplementary methods**

**1** **Immunofluorescence microscopy**

Cells on coverslips were washed with PBS, fixed in 4% PFA for 15 min, and permeabilized with 0.5% Triton X-100 (Sigma-Aldrich) for 30 min. The specified antibodies were thinned in PBS with 2% BSA and left to incubate at 4 °C overnight. The cells were washed with PBST (PBS with 2% Triton X-100) three times, followed by a 2-hour incubation with the secondary antibodies at room temperature in the dark. The images were acquired using Leica TCS SP8 STED microscope with a 63× silicon immersion lens. Additional antibody details are provided in the supplementary materials.

**2 Protein purification**

Various genetically modified proteins (His-OGT, His-NRF2, GST-NRF2, GST-NRF2-S103A, MBP-OGT, GST-OGT, GST-OGT-T444E and His-KEAP1) were expressed in *E. coli* BL21 (DE3) (Shenzhen Health Life Technology Co., Ltd.). The BL21 (DE3) cells were cultivated in LB medium at 37 °C and stimulated with 0.1 mM Isopropyl β-D-thiogalactopyranoside (Sigma-Aldrich) when OD_600_ reached 0.4-0.6, incubated overnight at 20 °C. The cells were lysed in a lysis solution containing 10 mM Na_2_HPO_4_, 1.8 mM KH_2_PO_4_ (pH 7.3), 140 mM NaCl, and 2.7 mM KCl, supplemented with of 0.1 mg/ml PMSF. To purify NRF2 with O-GlcNAcylation, the buffer was supplemented with 40 mM GlcNAc, 10 μM PUGNAc, 2 mM STZ, and 10  μM Thiamet G. For the purification of proteins with His-tag, the cell lysis supernatant was loaded onto the HisTrap HP column (Cytiva Life Science) and eluted with 50-200 mM imidazole (Sigma). For the purification of proteins with GST-tag, the cell lysis supernatant was loaded onto the GSTrap FF column (Cytiva Life Science) and eluted with 20 mM reduced glutathione (Sigma). For the purification of proteins with MBP-tag, the cell lysis supernatant was loaded onto the MBPTrap HP column (Cytiva Life Science) and eluted with 10 mM maltose (Sigma). The GST-NRF2 and MBP-OGT were co-expressed in the BL21(DE3) strain to express GST-NRF2 with O-GlcNAcylation. The recombinant NRF2 was purified by the GSTTrap HP column, and then the eluted proteins were loaded onto the MBPTrap HP column to remove the co-purified MBP-OGT. Centrifugal Filters Ultracel-30K (Millipore) were used to desalt and concentrate all the purified proteins.

**3 O-GlcNAc enzymatic labeling**

The cells were disrupted using O-GlcNAc lysis solution containing 20 mM HEPES (pH 7.9), 1% SDS, 1 mM Na_3_VO_4_, 10 mM NaF, 40 mM GlcNAc, 10 μM PUGNAc, 2 mM STZ, 10  μM Thiamet G, and full protease inhibitors. Endogenous NRF2 or Flag-NRF2 proteins were isolated from cell lysate by immunoprecipitation using anti-NRF2 antibody (Abcam) or anti-Flag M2 Affinity Gel (Sigma-Aldrich). Parallel control experiments were conducted without the presence of the labeling enzyme GalT1 Y289L. After isolating NRF2 using immunoprecipitation, it was labeled with an azido-containing nucleotide sugar analog (UDP-GalNAz) through enzymatic methods involving a modified (1,4)-galactosyltransferase (GalT1 Y289L) following the protocol of the Click-iT O-GlcNAc enzymatic labeling kit (Invitrogen, #C33368). Following labeling, the NRF2 protein was linked with an alkyne-biotin compound as per the instructions of the Click-iT protein analysis detection kit (Invitrogen, #C33372) protocol. Afterward, the samples labeled with biotin were isolated by the Streptavidin Magnetic Beads (Beaverbio, #22321) and detected by the Streptavidin-HRP or the specific antibody using immunoblotting.

**4 O-GlcNAc site mapping of NRF2**

The O-GlcNAc site mapping procedure is as previously reported [^1^](#_ENREF_1). Both His-NRF2 and MBP-OGT were simultaneously expressed in *E. coli* BL21(DE3) and the purified NRF2 was obtained using Ni-affinity chromatography [^2^](#_ENREF_2). The recombinant NRF2 protein band, separated by SDS-PAGE and stained with Coomassie blue R250 Stain (Bio-Rad), was then sent to PTM-Biolabs (Hang Zhou) Co., Ltd. Gel pieces for in-gel tryptic digestion were rinsed in 50 mM NH_4_HCO_3_ in 50% Acetonitrile (ACN) (v/v) until clear. Following that, the gel fragments were dried using 100 μl of pure ACN for 5 minutes, the excess liquid was eliminated, and the gel fragments were then soaked in 10 mM DTT and left at 56 °C for an hour. Subsequently, the gel fragments were once more dehydrated using pure ACN, the liquid was eliminated, and the gel fragments were then rehydrated with a solution containing 55 mM Iodoacetamide (IAA). The samples were incubated in darkness at room temperature for 45 minutes. Afterwards, the gel fragments were rinsed with a solution containing 50 mM NH_4_HCO_3_ and then dried using 100% acetonitrile. Afterward, the gel fragments were soaked in chymotrypsin solution (10 ng/μL) diluted in 50 mM NH_4_HCO_3_ on ice for a duration of 1 hour. After removing extra liquid, the gel pieces were then digested with chymotrypsin at 30 °C for the entire night. Peptides were extracted first with 50% ACN/5% Formic acid (FA), and then with 100% ACN. After drying, the peptides were reconstituted in a solution containing 2% acetonitrile and 0.1% formic acid. The tryptic peptides were ultimately dissolved in a solution containing 0.1% formic acid and then directly injected onto a reversed-phase analytical column for chromatographic separation. Peptide separation was carried out on a reversed-phase analytical column using a gradient starting at 4% and reaching 35% solvent B (0.1% formic acid in 98% acetonitrile) within 22 minutes. This was followed by a 35% to 80% increase in 5 minutes, and maintained at 80% for the final 3 minutes, all at a steady flow rate of 400 nL per minute on an EASY-nLC 1000 UPLC system. Next, the peptides underwent NSI source before being analyzed using tandem mass spectrometry (MS/MS) in Q ExactiveTM plus (Thermo) connected online to the UPLC. Complete peptides were identified in the Orbitrap with a precision of 70,000, whereas ion fragments were identified in the Orbitrap with a precision of 17500.A method based on data was used, switching between a single MS scan and 20 MS/MS scans for the top 20 precursor ions with an ion count above 5E3 in the MS survey scan, and a dynamic exclusion of 15.0 seconds. A voltage of 2.0 kV was applied for electrospray, with automatic gain control (AGC) utilized to avoid overfilling the orbitrap with 5E4 ions collected for generating MS/MS spectra. The mass spectrometry scans ranged from m/z 350 to 1800.The MS/MS data obtained were analyzed with the Mascot search engine (v.2.3.0), searching tandem mass spectra against the target sequences database. Chymotrypsin was specified as the cleavage enzyme with allowance for up to 4 missing cleavages. A tolerance of 10 parts per million was established for precursor ions and 0.02 Daltons for fragment ions, with O-GlcNAc on Ser/Thr designated as a variable alteration. A minimum peptide ion score of 20 was established.

**5 GST pull-down assay**

The purified proteins (300 ng) or GST protein (200 ng) was incubated with glutathione Sepharose 4B beads (Cytiva Life Science) in NP40 lysis buffer overnight at 4 °C. After being washed five times with NP40 lysis buffer, the protein on the beads were analyzed using immunoblotting.

**6 Luciferase reporter assay**

Transfection of the cells involved introducing 1 μg of a pARE-luc luciferase reporter plasmid and 0.1 μg of Renilla luciferase transfection control (Promega) using Attractene transfection reagent (Qiagen). Following a 24-hour transfection period, the cells were lysed and quantified with a dual-luciferase assay (Promega) using an EnSpire multimode plate reader (PerkinElmer). The expression of luciferase dependent on ARE was standardized based on the Renilla luciferase measurements.

**7 *In vivo* ubiquitination assays**

The ubiquitination test was carried out as previous study [^3^](#_ENREF_3). His-Ubiquitin and HA-NRF2 were transfected into NCI-H1299 cells when they reached 80% confluence. After transfecting cells for 36 hours, they were then treated with the specified compounds. Following the treatment, the cells were lysed using buffer A containing 6 M guanidine-HCl, 0.1 M Na_2_HPO_4_/NaH_2_PO_4_, and 10 mM imidazole at pH 8.0. After sonication, the lysates were mixed with nickel-nitrilotriacetic acid (Ni-NTA) beads from Cytiva Life Science and left at room temperature for 3 hours. Afterward, His-tagged proteins were washed in the following order: buffer A (twice), buffer A/TI (twice), and buffer TI (once). Buffer A consists of 1 volume buffer A and 3 volumes buffer TI, while buffer TI contains 25 mM Tris-HCl and 20 mM imidazole at pH 6.8. Finally, the proteins pulled down were separated using SDS-PAGE for immunoblot analysis.

**8 Quantitative reverse transcription PCR (RT-qPCR)**

RNA samples were extracted with the QIAGEN RNeasy mini kit, followed by reverse-transcribed using the HiScript III 1st Strand cDNA Synthesis Kit (Vazyme Biotech Co., Ltd.). The cDNA obtained in the above step was mixed with primers and the QuantiNova SYBR Green PCR Kit (Qiagen). Subsequently, the ABI-7500 Real-time PCR system was utilized to conduct the reactions. The normalized target gene expression was evaluated by 2^-ΔΔCt^ using β-actin as reference gene. The primer was synthesized by RuiBoBio (Qingdao, China), as shown in the Table S1.

**9 Cell viability assay**

The cells were seeded in 96-well plates. At certain times, the cells were incubated with the MTT solution (Yeasen, #40206ES76) at 37 °C for 4 hours. After discarding the medium, the formazan crystals were dissolved in DMSO (Sigma-Aldrich), and the absorbance was then measured by microplate reader at 490 nm.

**10 Colony-formation assay**

400 cells were seeded per well of 6-well plates. Following a growth period of 10-14 days, the colonies were fixed with 4% PFA, dyed with crystal violet (0.5% w/v) (Solarbio), and then captured in photographs.

**11 Transwell cell migration assay**

Cells were trypsinized, then suspended in a medium without FBS, and 3×10^4^ finally placed in the upper chamber of a Transwell with 8 mm pore size (Corning). The well was filled with culture medium. Following the elimination of stationary cells, the cells that moved across the membrane were fixed with 4% PFA, dyed with 0.5% crystal violet, and evaluated through photography.

**12 Cell cycle assay**

Cell cycle analysis was conducted using the Cell Cycle Analysis Kit (Yeasen, #40301ES50) following the manual. Briefly, cells were trypsinized and washed with cold PBS. Cell was fixed with pre-cooled 70% ethanol for over 2 hours at -20 °C. Washed with PBS, the cells were resuspended into 0.5 mL staining buffer mixed with 10 μL propidine iodide (PI) solution and 10 μL of RNase A solution. After cultured at 37°C in the dark for 30 minutes, the cells were analyzed by flow cytometry after being filtered through screen.

**13 Cell apoptosis detection**

Cell apoptosis was assessed using the Annexin V-FITC/PI apoptosis detection kit (Yeasen, #40302ES20). Briefly, cells were trypsinized with trypsin (without EDTA), and collected. Rinse the cells two times with chilled PBS at 4°C, then spin at 300 g for 5 minutes. Washed with PBS, the cells were resuspended into 250 μL binding buffer mixed with 5 μL of Annexin V-FITC and 10 μL of propidine iodide (PI). After cultured at room temperature in the dark for 15 minutes, the cells were added 400 μL PBS and analyzed by flow cytometry.

**14 Reactive oxygen species assay**

DCFH-DA (Beyotime, #S0033S) was diluted with the serum-free medium at 1:1000 and the final concentration was 10 mmol/L. The cells were cultured with DCFH-DA working solution at 37°C in the dark for 20 minutes. Then the cells were washed thrice using a serum-free medium. After treatment with indicated compounds, the fluorescence intensity was measured using a microplate reader.

**References**

1. Han C, Gu Y, Shan H, et al. O-GlcNAcylation of SIRT1 enhances its deacetylase activity and promotes cytoprotection under stress. *Nature Communications*. 2017/11/14 2017;8(1):1491. doi:10.1038/s41467-017-01654-6

2. Gao H, Shi M, Wang R, et al. A widely compatible expression system for the production of highly O-GlcNAcylated recombinant protein in Escherichia coli. *Glycobiology*. Dec 1 2018;28(12):949-957. doi:10.1093/glycob/cwy077

3. Zhang J, Bu X, Wang H, et al. Cyclin D-CDK4 kinase destabilizes PD-L1 via cullin 3-SPOP to control cancer immune surveillance. *Nature*. Jan 4 2018;553(7686):91-95. doi:10.1038/nature25015

| Table S1 The sequence of primers | |
| --- | --- |
| primers | sequences |
| NRF2-F | AACCAGTGGATCTGCCAACTACTC |
| NRF2-R | CTGCGCCAAAAGCTGCAT |
| HO-1 -F | TGCTCAACATCCAGCTCTTTGA |
| HO-1 -R | GCAGAATCTTGCACTTTGTTGCT |
| GCLM-F | GCTGTATCAGTGGGCACAG |
| GCLM-R | CGCTTGAATGTCAGGAATGC |
| NQO1-F | ATTGAATTCGGGCGTCTGCTG |
| NQO1-R | AGGCTGGTTTGAGCGAGT |
| β-actin -F | CTGGAACGGTGAAGGTGACA |
| β-actin -R | AAGGGACTTCCTGTAACAATGCA |

**
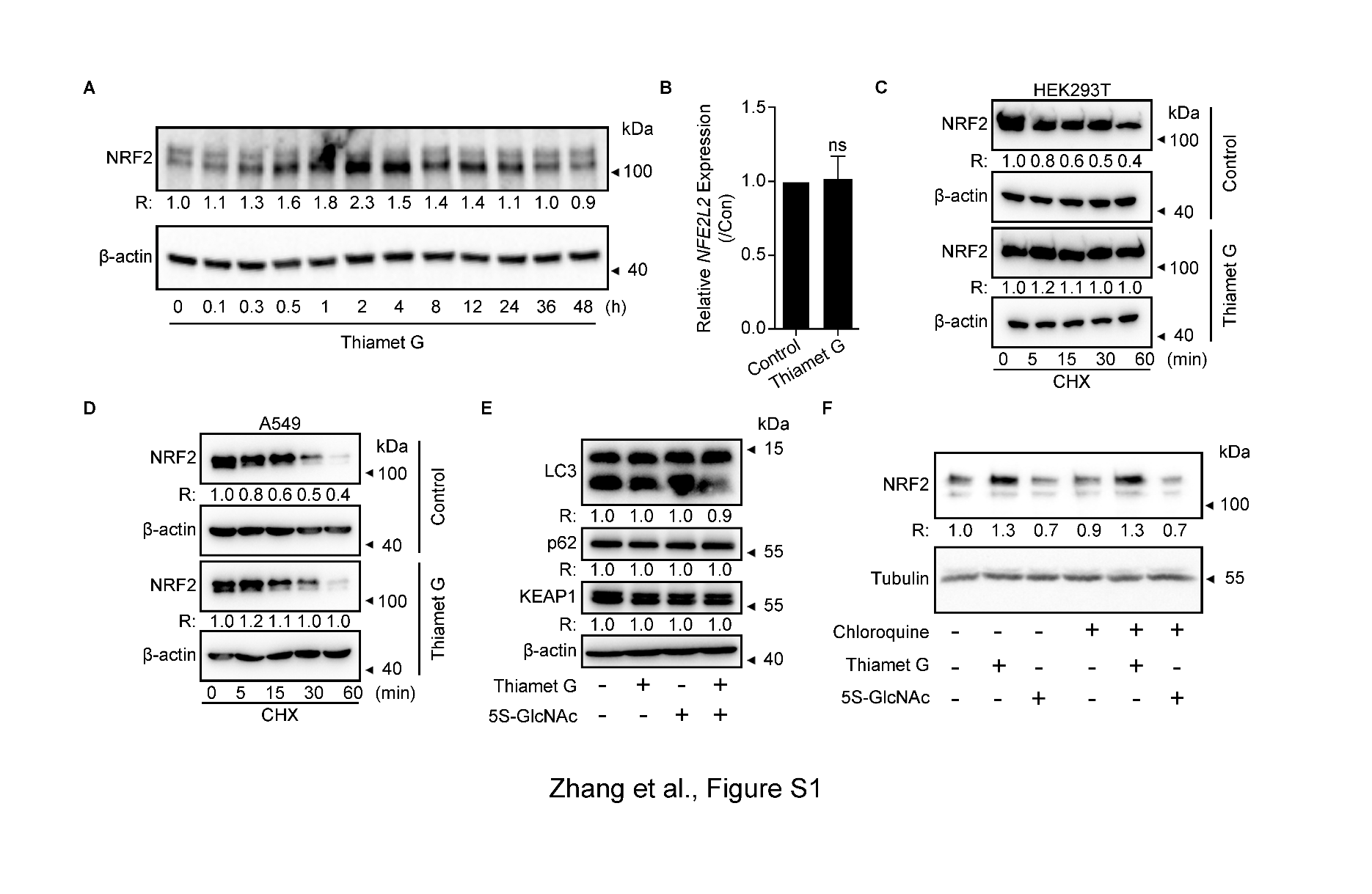
**

**Figure S1. Assessment for the effect of global O-GlcNAcylation on NRF2 expression, related to Figure 1.**

(A) The expression of NRF2 was detected by WB in NCI-H1299 cells, which were treated with 2 μM Thiamet G and incubated for the indicated durations. Data represent three independent experiments.

(B) The expression of *NFE2L2* was measured by real-time RT-PCR in NCI-H1299 cells (Data represent three independent experiments, ns, not significant, using unpaired two-tailed Student’s *t*-test).

(C) HEK293T cells were pretreated with Thiamet G (2 μM) for 2 h, treated with 2 μg/mL CHX and incubated for the indicated durations. Data represent two independent experiments.

(D) A549 cells were pretreated with Thiamet G (2 μM) for 2 h, treated with 2 μg/mL CHX and incubated for the indicated durations. Data represent two independent experiments.

(E) LC3, p62 and KEAP1 expression in NCI-H1299 cells treated with Thiamet G (2 μM) or/and 5S-GlcNAc (50 μM) for 2 h was probed by WB. Data represent two independent experiments.

(F) NRF2 protein levels were determined by WB in indicated treatment for 2 h. (Thiamet G, 2 μM; 5S-GlcNAc, 50 μM; chloroquine, 10 μM) Data represent two independent experiments.

The relative intensities of proteins in immunoblotting were determined by normalizing the intensities of corresponding proteins to the intensities of β-actin.

**
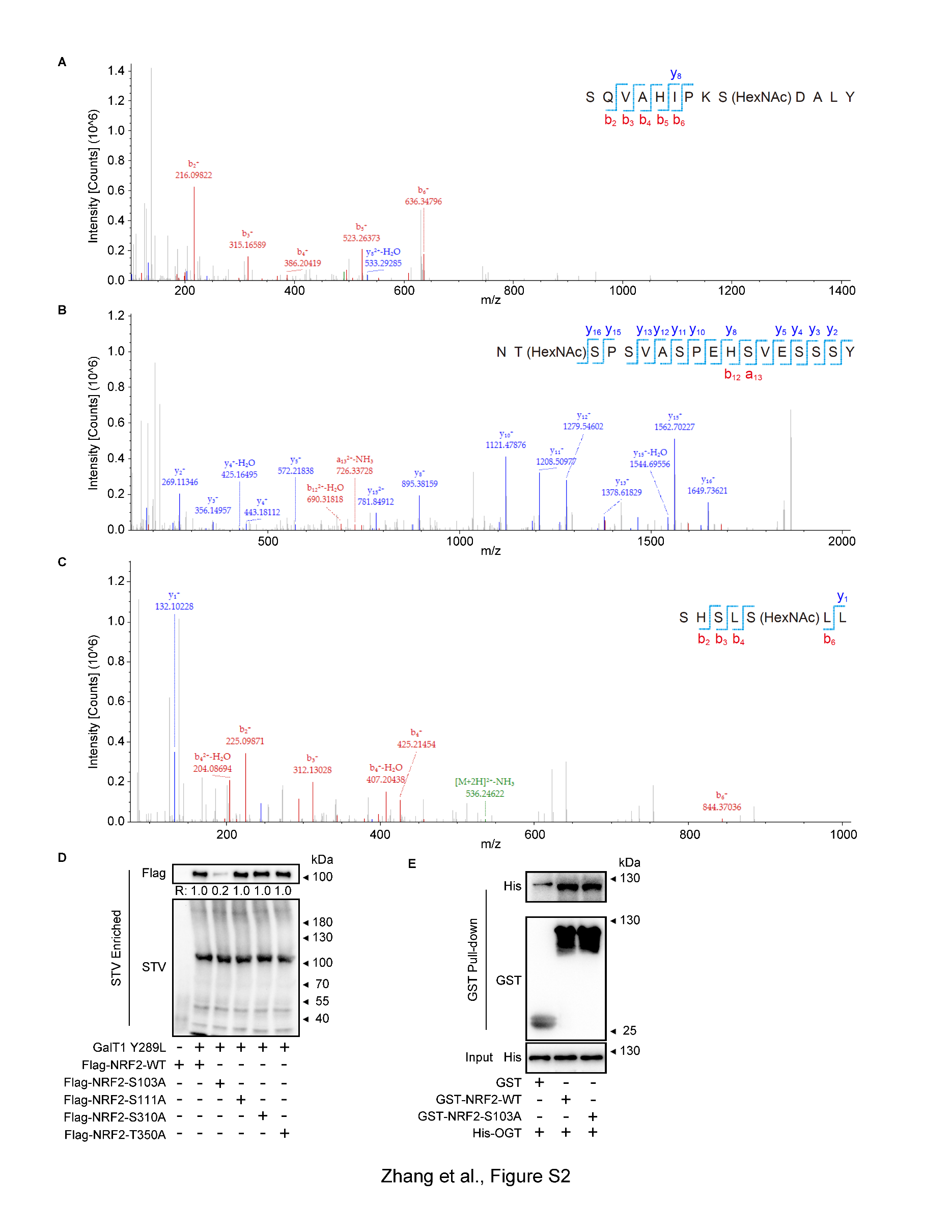
**

**Figure S2. Identification of O-GlcNAcylated sites in NRF2, related to Figure 2.**

(A-C) LC-MS/MS detected the O-GlcNAcylated sites of NRF2 (Ser111, A; Ser310, B; T350, C).

(D) Flag-tagged NRF2^WT^, NRF2^S103A^, NRF2^S111A^, NRF2^S310A^ or NRF2^T350A^ vector was transfected into NCI-H1299 cells. The cells were lysed after treatment with 2 μM Thiamet G for 2 h and labeled by chemoenzymatic labeling. And then the biotinylated proteins were enriched and probed with NRF2 antibody. Data represent two independent experiments.

(E) Direct interaction of OGT with NRF2^WT^ and NRF2^S103A^ in an *in vitro* GST pull-down assay. Data represent two independent experiments.

The relative intensities of proteins in immunoblotting were determined by normalizing the intensities of corresponding proteins to the intensities of β-actin.

**
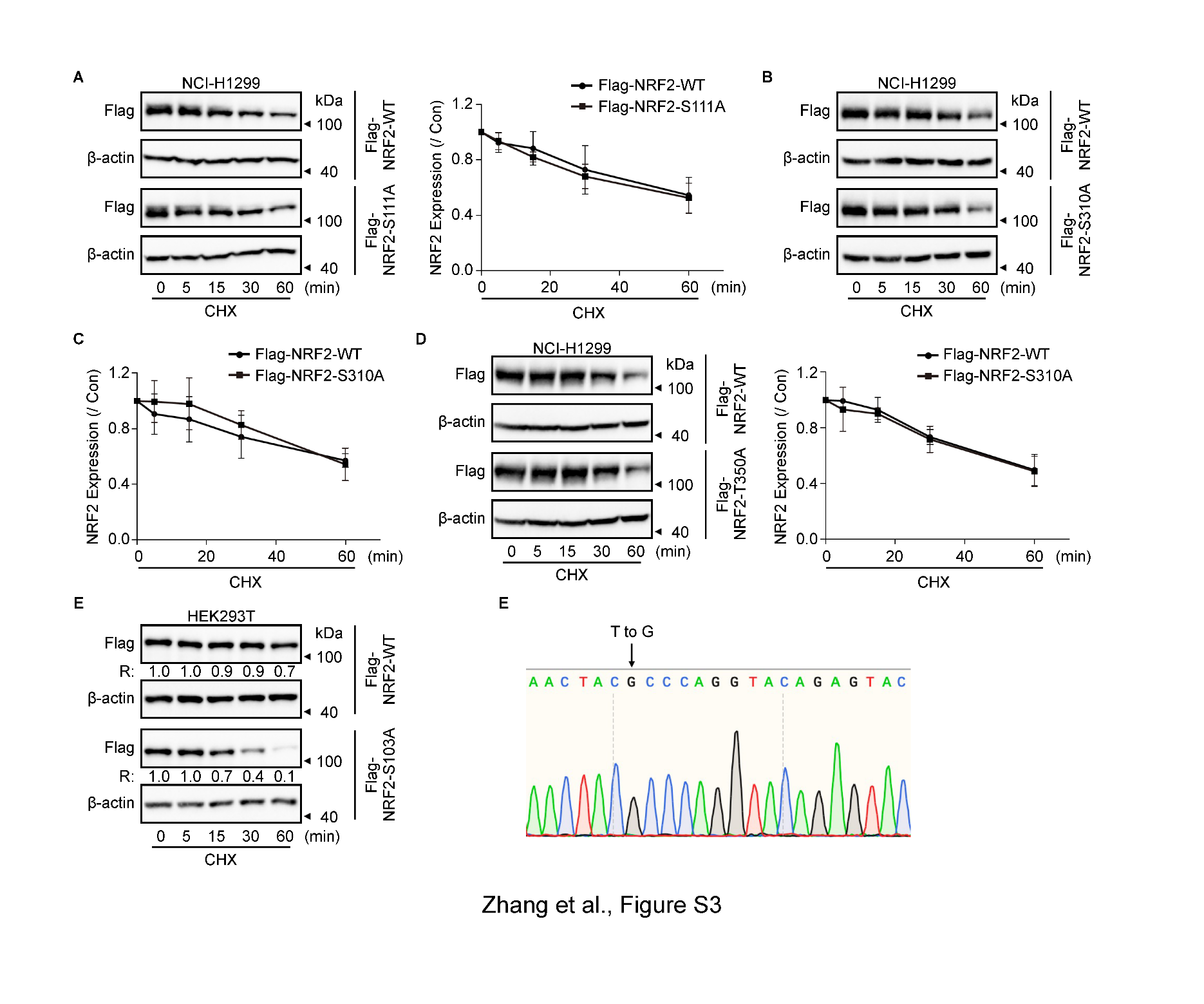
**

**Figure S3. Assessment for the effects of NRF2 O‐GlcNAcylation sites on its stability, related to Figure 3.**

(A-D) The stability of NRF2 was determined by WB in NCI-H1299 cells which were translated Flag-tagged NRF2^S111A^ (A), NRF2^S310A^ (B-C) or NRF2^T350A^ (D) in the presence of CHX for the indicate time periods. Data represent three independent experiments.

(E) The stability of NRF2 was determined by WB (probed with anti-Flag antibody) in HEK293T cells which were translated Flag-tagged NRF2^WT^ or NRF2^S103A^ in the presence of CHX for the indicate time periods. Data represent three independent experiments.

(F) PCR products were sequenced to identify *NFE2L2* mutation (307T→G, S103A) using CRISPR/Cas9 gene editing.

The relative intensities of proteins in immunoblotting were determined by normalizing the intensities of corresponding proteins to the intensities of β-actin.

**
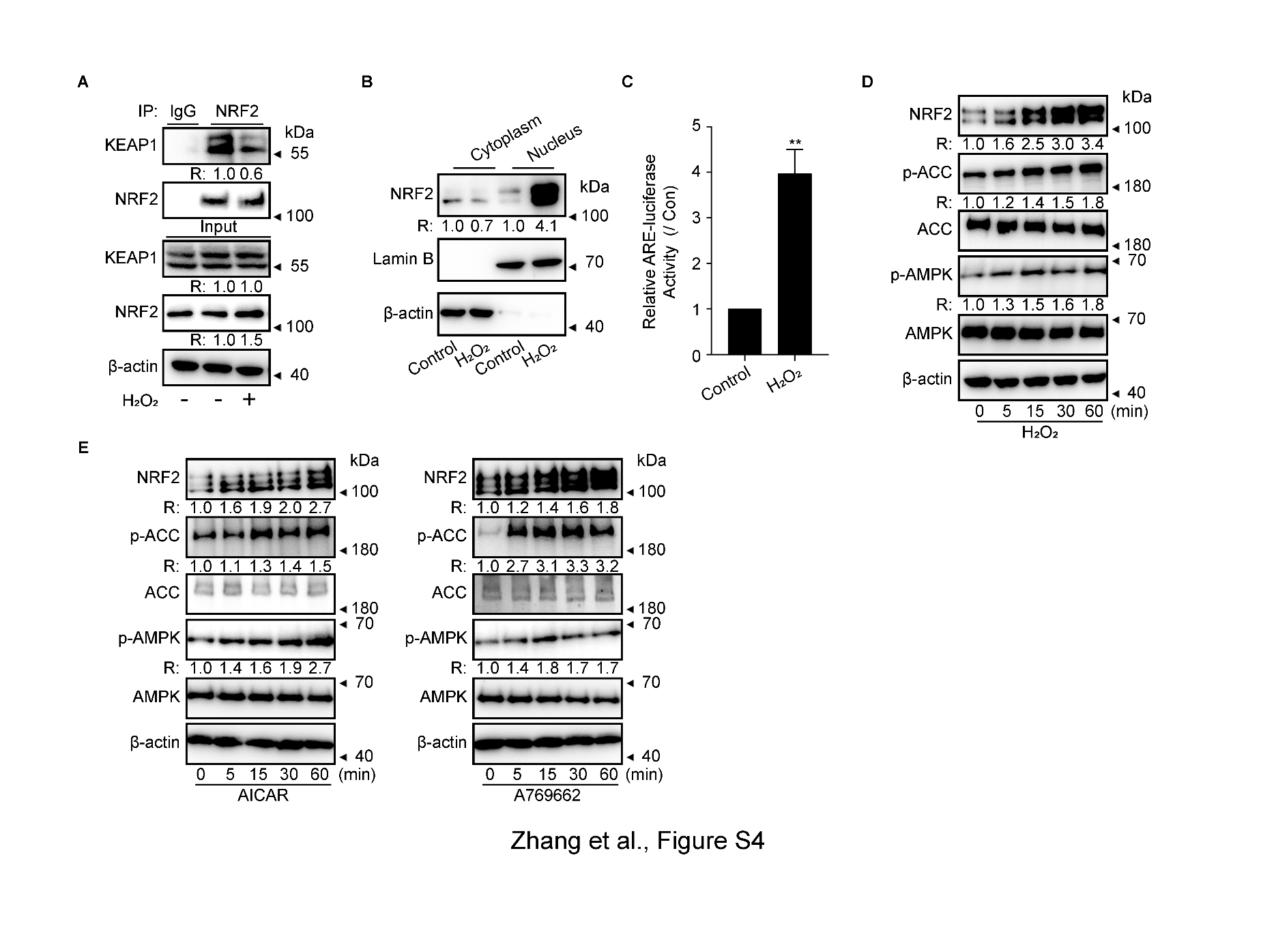
**

**Figure S4. Measurement for NRF2 activation and AMPK/OGT pathway upon H2O2 stimulation, related to Figure 4.**

(A) Interactions between KEAP1 and NRF2 was determined by Co-IP assay using in NCI-H1299 cells treated with H_2_O_2_ (50 μM) for 45 min. Data represent three independent experiments.

(B) The distribution of NRF2 was determined by nuclear and cytoplasmic fraction in NCI-H1299 cells treated with H_2_O_2_ (50 μM) for 45 min. Data represent three independent experiments.

(C) NCI-H1299 cells were transfected with the ARE luciferase reporter and at 24 h post-transfection, the cells were treated with H_2_O_2_ (50 μM) for 45 min and then analyzed for luciferase activity (Data represent three independent experiments, ***P* < 0.01, using unpaired two-tailed Student’s *t*-test).

(D) The expression of p-ACC, ACC, p-AMPK, AMPK and NRF2 were measured by WB in NCI-H1299 cells with H_2_O_2_ (50 μM) for the indicate time periods. Data represent three independent experiments.

(E) The expression of p-ACC, ACC, p-AMPK, AMPK and NRF2 were measured by WB in the presence of AICAR (1 mM) or A-769662 (50 μM) for the indicate time periods. Data represent three independent experiments.

The relative intensities of proteins in immunoblotting were determined by normalizing the intensities of corresponding proteins to the intensities of β-actin.

**
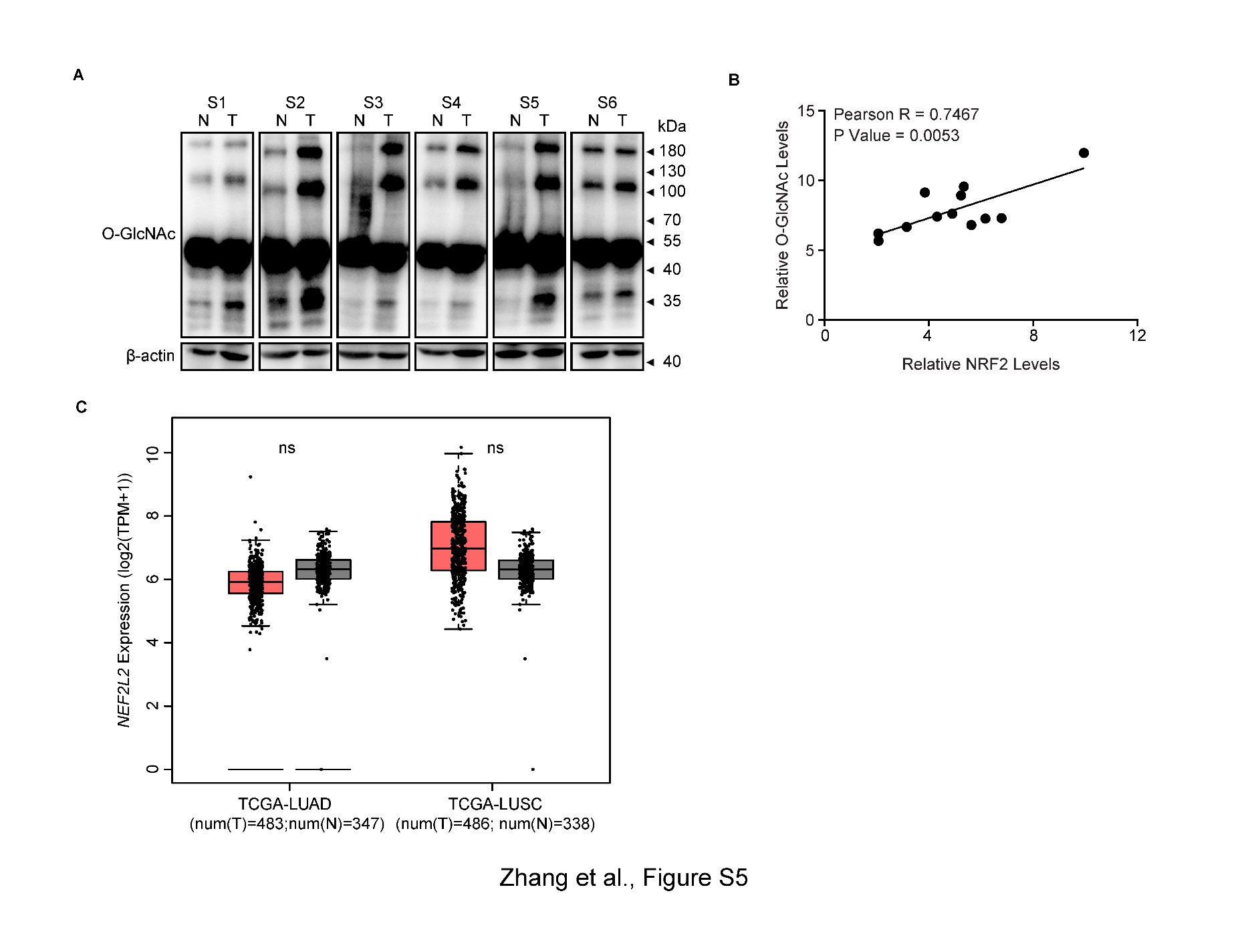
**

**Figure S5. Correlation analysis between global O-GlcNAcylation and NRF2 in 6 cases of human lung cancer, related to Figure 5.**

(A) Representative WB of global O-GlcNAcylation in 6 human lung cancer (T) and paired adjacent normal controls (N).

(B) Correlation analysis between NRF2 expression and global O-GlcNAcylation (n = 6). The relative intensities of proteins in immunoblotting were determined by normalizing the intensities of corresponding proteins to the intensities of β-actin.

(C) The *NFE2L2* expression analysis from TCGA database in GEPIA. (LUAD = Lung adenocarcinoma, LUSC = Lung squamous cell carcinoma, T = Tumor, N = Normal)
